# Anxiety and behavioral changes in Japanese patients with inflammatory bowel disease due to COVID-19 pandemic: a national survey

**DOI:** 10.1007/s00535-022-01949-6

**Published:** 2023-01-06

**Authors:** Hiroshi Nakase, Kohei Wagatsuma, Masanori Nojima, Takayuki Matsumoto, Minoru Matsuura, Hideki Iijima, Katsuyoshi Matsuoka, Naoki Ohmiya, Shunji Ishihara, Fumihito Hirai, Ken Takeuchi, Satoshi Tamura, Fukunori Kinjo, Nobuhiro Ueno, Makoto Naganuma, Kenji Watanabe, Rintaro Moroi, Nobuaki Nishimata, Satoshi Motoya, Koichi Kurahara, Sakuma Takahashi, Atsuo Maemoto, Hirotake Sakuraba, Masayuki Saruta, Keiichi Tominaga, Takashi Hisabe, Hiroki Tanaka, Shuji Terai, Sakiko Hiraoka, Hironobu Takedomi, Kazuyuki Narimatsu, Katsuya Endo, Masanao Nakamura, Tadakazu Hisamatsu

**Affiliations:** 1 Department of Gastroenterology and Hepatology, Sapporo Medical University School of Medicine, S-1, W-16, Chuo-Ku, Sapporo, Hokkaido, 060-8543 Japan; 2 Center for Translational Research, The Institute of Medical Science, The University of Tokyo, Minato-Ku, Japan; 3 Division of Gastroenterology, Department of Internal Medicine, Iwate Medical University, Shiwa, Japan; 4 Department of Gastroenterology and Hepatology, Kyorin University School of Medicine, Mitaka, Japan; 5 Department of Gastroenterology and Hepatology, Osaka University Graduate School of Medicine, Suita, Japan; 6 Division of Gastroenterology and Hepatology, Department of Internal Medicine, Toho University Sakura Medical Center, Sakura, Japan; 7 Departments of Gastroenterology and Advanced Endoscopy, Fujita Health University School of Medicine, Toyoake, Japan; 8 Department of Internal Medicine II, Faculty of Medicine, Shimane University, Izumo, Japan; 9 Department of Gastroenterology, Faculty of Medicine, Fukuoka University, Fukuoka, Japan; 10 Department of Gastroenterology, IBD Center, Tsujinaka Hospital Kashiwanoha, Kashiwa, Japan; 11 First Department of Medicine, Hamamatsu University School of Medicine, Hamamatsu, Japan; 12 Center for Gastroenterology, Urasoe General Hospital, Urasoe, Japan; 13 Division of Metabolism and Biosystemic Science, Gastroenterology, and Hematology/Oncology, Asahikawa Medical University, Asahikawa, Japan; 14 Department of Medicine, Asahikawa Medical University, Asahikawa, Japan; 15 The Third Department of Internal Medicine, Kansai Medical University Hirakata, Hirakata, Japan; 16 Center for Inflammatory Bowel Disease, Division of Internal Medicine, Hyogo College of Medicine, Nishinomiya, Japan; 17 Division of Gastroenterology, Tohoku University Hospital, Sendai, Japan; 18 Department of Gastroenterology, Sameshima Hospital, Kagoshima, Japan; 19 IBD Center, Sapporo Kosei General Hospital, Sapporo, Japan; 20 Division of Gastroenterology, Matsuyama Red Cross Hospital, Matsuyama, Japan; 21 Department of Gastroenterology, Kagawa Prefectural Central Hospital, Takamatsu, Japan; 22 Inflammatory Bowel Disease Center, Sapporo Higashi Tokushukai Hospital, Sapporo, Japan; 23 Department of Gastroenterology and Hematology, Hirosaki University Graduate School of Medicine, Hirosaki, Japan; 24 Department of Gastroenterology and Hepatology, Division of Internal Medicine, The Jikei University School of Medicine, Minato-Ku, Japan; 25 Department of Gastroenterology, Dokkyo Medical University, Shimotsuga, Japan; 26 Department of Gastroenterology, Fukuoka University Chikushi Hospital, Chikushino, Japan; 27 Sapporo IBD Clinic, Sapporo, Japan; 28 Division of Gastroenterology, Graduate School of Medical and Dental Sciences, Niigata University, Niigata, Japan; 29 Department of Gastroenterology and Hepatology, Dentistry and Pharmaceutical Sciences, Okayama University Graduate School of Medicine, Okayama, Japan; 30 Division of Gastroenterology, Department of Internal Medicine, Faculty of Medicine, Saga University, Saga, Japan; 31 Department of Internal Medicine, National Defense Medical College, Tokorozawa, Japan; 32 Division of Gastroenterology, Tohoku Medical and Pharmaceutical University, Sendai, Japan; 33 Department of Gastroenterology and Hepatology, Nagoya University Graduate School of Medicine, Nagoya, Japan

**Keywords:** Inflammatory bowel disease, COVID-19, Questionnaire survey, Anxiety

## Abstract

**Background:**

Given the increasing health concerns for patients with inflammatory bowel disease (IBD), amidst the COVID-19 pandemic, we investigated the impact of the pandemic on the anxiety and behavioral changes in Japanese patients with IBD.

**Methods:**

We analyzed 3032 questionnaires from patients with IBD, aged 16 years or older visiting 30 hospitals and 1 clinic between March 2020 and June 2021. The primary outcome was the score of the anxiety experienced by patients with IBD during the pandemic.

**Results:**

Participants reported a median age of 44 years; 43.3% of the patients were women. Moreover, 60.6% and 39.4% were diagnosed with ulcerative colitis and Crohn’s disease, respectively, with a median disease duration of 10 years. Participants indicated an average of disease-related anxiety score of 5.1 ± 2.5 on a ten-point scale, with a tendency to increase, 1 month after the number of infected persons per population increased. The top three causes for anxiety were the risk of contracting COVID-19 during hospital visits, SARS-CoV-2 infection due to IBD, and infection by IBD medication. Factors associated with anxiety were gender (women), being a homemaker, hospital visit timings, mode of transportation (train), use of immunosuppressive drugs, and nutritional therapy. Most patients continued attending their scheduled hospital visits, taking their medications, experienced the need for a family doctor, and sought guidance and information regarding COVID-19 from primary doctors, television, and Internet news.

**Conclusions:**

Patients with IBD experienced moderate disease-related anxiety due to the pandemic and should be proactively informed about infectious diseases to relieve their anxiety.

**Supplementary Information:**

The online version contains supplementary material available at 10.1007/s00535-022-01949-6.

## Introduction

After the rapid spread of the novel coronavirus disease 2019 (COVID-19), the World Health Organization declared it a global pandemic on March 11, 2020 [1]. Increasing evidence indicates that older and immunocompromised patients, and those with chronic diseases, are at a higher risk of contracting severe or fatal COVID-19 [2].

Inflammatory bowel disease (IBD) comprises two major disease types—ulcerative colitis (UC) and Crohn's disease (CD), which occur at a relatively young age with a high incidence rate between late teens and early 30s [3]. Patients with IBD endure the disease throughout their lives. Therefore, it is important to maintain patients’ quality of life (QOL) by focusing on treatment alongside their overall life. Owing to the COVID-19 pandemic, global citizens, including those in Japan, are restricting unnecessary movement outside the house, and many patients with stable symptoms are postponing medical visits or being prescribed medication over the phone [4, 5].

Patients with IBD experience repeated relapses; subsequently, they undergo major dilemmas between the threat of contracting COVID-19 by visiting the doctor and the anxiety of disease progression by postponing the visit [6, 7]. Additionally, steroids, immunomodulators, and biologics that influence the risk of COVID-19 are often used in the treatment of patients with IBD, but the extent of their influence is still under investigation. Therefore, it is possible that these patients are unaware about how to obtain COVID-19-related information and may have voluntarily reduced or withdrawn their medication. Moreover, since health-care providers were not entirely familiar with COVID-19 themselves, it is unclear how much information was provided to the patients regarding daily life practices and risks related to COVID-19. Several questionnaire surveys on the anxiety of patients with IBD during the pandemic demonstrated the impact of COVID-19 on health and psychological well-being alongside the increasing concern regarding the risk of contracting COVID-19 and medication continuation [6–13].

Meanwhile, significant difference in these factors owing to regional and national characteristics, and medical conditions have been indicated. However, no such large-scale survey has been conducted in Japan. Therefore, this study conducted a questionnaire survey to investigate the anxiety and behavioral changes among Japanese patients with IBD during the COVID-19 pandemic. This study was referred to as the Japan COVID-19 Survey and the Questionnaire for Inflammatory Bowel Disease (J-DESIRE).

## Methods

### Patient recruitment

At each recruiting institution, the attending physician personally explained the aim and contents of the survey to each patient with IBD, and asked them to complete a paper-based questionnaire related to COVID-19, after obtaining their written informed consent. Within 2 weeks of distribution, the completed questionnaires were sent to the Department of Gastroenterology, at the Sapporo Medical University Hospital. The participant recruitment period was between March 2020 and June 2021.

### Questionnaire

First, the co-authors developed the questionnaire by referring to previous reports [8, 14] and adding questions that they thought would be useful for gathering information regarding anxiety of IBD patients. After that, Japan IBD COVID-19 task force members (HN, TM, MM, HI, KM, NO, SI, FH, and TH) conducted internal evaluations several times to complete the questionnaire. The questionnaire consisted of six major domains, some of which were rated on a ten-point visual analog scale (VAS) and focused on the impact of COVID-19 on anxiety related to IBD, disease activity, medical examination, IBD medication, and prevention of infection (Questionnaire in the Supplementary Material). The reason for using the VAS in this study was to ensure that patients could show their feelings easily and the difference could be measured. Additionally, we collected and assessed data regarding demographics, socioeconomic status, IBD diagnoses, comorbidities, and current IBD treatment. The primary outcome was the VAS score for disease-related anxiety during the pandemic [Q1. (1) How much anxiety did you feel about your disease due to the outbreak of SARS-CoV-2?]. This study protocol was approved by the institutional review board of Sapporo Medical University and registered publicly on the UMIN registration (No. UMIN000041191).

### Statistical analysis

Descriptive statistics were computed using mean and standard deviation (SD) for continuous variables—ten-point VAS scores—and the proportion for categorical variables.

Two or more continuous variables groups were compared using Student’s *t* test or analysis of variance (ANOVA). Pearson's correlation was used to examine the relationship between the number of infections per population (in the current month, 1 month ago, and 2 months ago) and anxiety scores. Univariate and multivariate analyses were exploratorily performed using general linear models to assess the factors associated with the primary outcome (VAS score of disease-related anxiety) with/without adjusting for potential confounding factors. We calculated variance inflation factor (VIF) to evaluate multicollinearity for included covariates, and considered it acceptable if VIF < 5 for each coefficient to be interpreted.

Statistical significance was set at *P* < 0.05. All analyses were performed using SPSS version 25 (IBM, Armonk, NY, USA). Due to the descriptive and exploratory nature of this epidemiological study, we employed the convenience sampling method for participant recruitment; therefore, sample size was not calculated using power tests. Based on the current sample size and the parameters obtained a posteriori (standard deviation: 2.5), a difference of 1.3 can be detected in the VAS scores for disease-related anxiety between the two equal-size groups with a probability of 91% (Student's *t* test). This probability is 81%, if the ratio of the group size is 1:3. No imputations were performed for missing data, and those with complete data for entered items were included in the univariate and multivariate analyses, respectively.

## Results

### Demographics and clinical characteristics

A total of 3790 questionnaires were distributed with a return rate of 80.4% (3049/3790). Of these, 17 were excluded because of withdrawal of consent. Finally, 3032 questionnaires were analyzed. Tables 1 and 2 present the participants’ demographic data. Descriptive analyses revealed that 43.3% were women and participants’ median age was 44 years (ranging: 16–92 years) (Fig. S1). Moreover, 60.6% and 39.4% had been diagnosed with UC and CD, respectively. The analysis of patients' hospital visits revealed that only 7.1% of the patients visited community doctors near their homes, while 92.9% of the patients visited a specialized IBD facility. The study period spanned from the middle of the second pandemic wave to the end of the four waves of COVID-19 in Japan (Fig. S2). Fig. S3 presents the number of questionnaires collected by region.

**Table 1:** Participants’ demographic characteristics at baseline

Characteristic	n = 3032† n (%)
Age (years)	M = 44 (IQR = 16–92)
Female sex—no. (%)	1311/3030 (43.3)
Married—no. (%)	1229/3009 (40.8)
Co-resident—no. (%)	2525/3013 (83.8)
Occupation—no. (%)	
Student	154 (5.1)
Part-time job	431 (14.3)
Company employee	1346 (44.7)
Civil servant	212 (7.0)
Self-employed	212 (7.0)
Homemaker	287 (9.5)
Unemployed	366 (12.2)
Disease—no. (%)	
Ulcerative colitis	1817/2998 (60.6)
Crohn's disease	1181/2998 (39.4)
Medical history (years)— no. (%)	
≦5.00	983/2973 (33.1)
5.01–10.00	654/2973 (22.0)
10.10–15.00	427/2973 (14.4)
15.01–20.00	322/2973 (10.8)
≧20.01	587/2973 (19.7)
Surgical history (times) — no. (%)	
≦0	2070/2865 (72.3)
1	359/2865 (12.5)
2	198/2865 (6.9)
3–5	209/2865 (7.3)
6–10	24/2865 (0.8)
≧11	5/2865 (0.2)
Stoma (artificial anus) — no. (%)	145/2973 (4.9)
Self-assessment of disease activity using VAS — no. (%)	
Remission (VAS 1)	1134/3007 (37.7)
Mild (VAS 2–4)	1071/3007 (35.6)
Moderate (VAS 5–7)	581/3007 (19.3)
Severe (VAS 8–10)	221/3007 (7.3)
Visiting a hospital near home for treatment of ulcerative colitis or Crohn's disease—no. (%)	213/2982 (7.1)
Usual interval between visits to the hospital (months) — no. (%)	
≦1	776/2987 (26.0)
2–3	2196/2987 (73.5)
≧4	15/2987 (0.5)
Time from home to hospital (h) — no. (%)	
≦0.5	1501/2992 (40.2)
1–2	1428/2992 (47.7)
≧3	63/2992 (2.1)
Usual means of commuting to the hospital— no. (%)‡	
Walking or bicycling	233/3005 (7.8)
Car	2180/3005 (72.5)
Bus	315/3006 (10.5)
Train	604/3006 (20.1)
Others	80/2814 (2.8)

IQR: interquartile range, VAS: visual analog scale

^†^ Missing values for each item were excluded from the denominator

^‡^ There is duplication

**Table 2:** Current treatment status for inflammatory bowel disease

Current treatment status of you†	n (%)
5-Aminosalicylic acid preparations	2338/3005 (79.5)
Pentasa suppositories	361/3005 (12.0)
SASP suppositories	17/3005 (0.6)
5-ASA enema	134/3005 (4.5)
Oral steroid	208/3005 (6.9)
Parenteral steroid	37/3005 (1.2)
Steroid suppositories	23/3005 (0.8)
Steroid enema (STERONEMA®)	9/3005 (0.3)
Steroid enema (PREDONEMA®)	19/3005 (0.6)
Oral budesonide	50/2814 (1.8)
Budesonide enema	195/3005 (6.5)
Thiopurine	944/3005 (31.4)
Tacrolimus	29/3005 (1.0)
Infliximab	574/3005 (19.1)
Adalimumab	385/3005 (12.8)
Golimumab	68/3005 (2.3)
Ustekinumab	252/3005 (8.4)
Vedolizumab	178/3005 (5.9)
Tofacitinib	72/3005 (2.4)
Granulocyte apheresis therapy	34/3005 (1.1)
Nutritional therapy	410/2813 (14.6)

^†^ There is duplication

### Changes in and contents of patients’ anxiety during COVID-19 pandemic

**Fig. 1:**
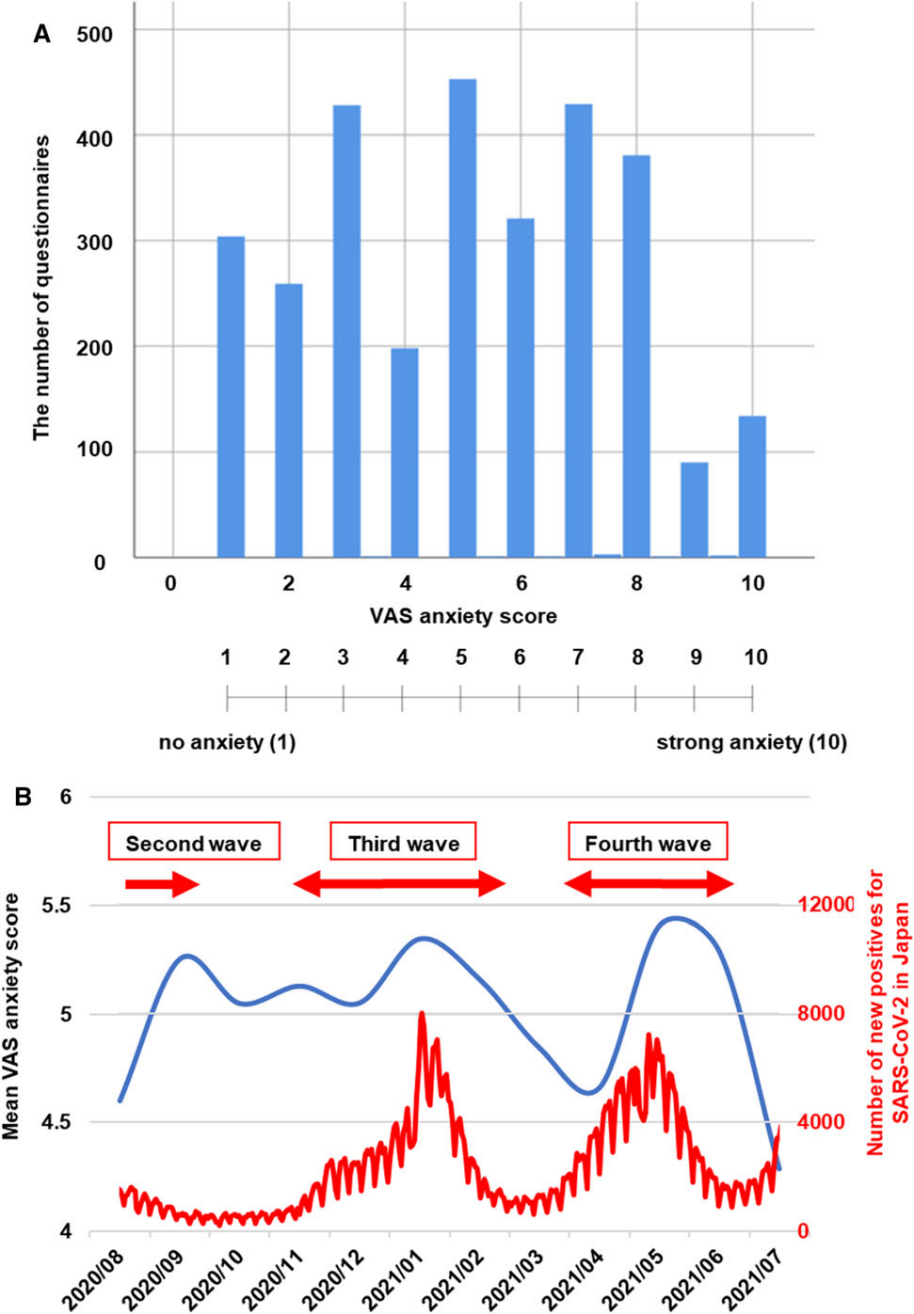
VAS scores for disease-related anxiety during the COVID-19 pandemic. (A) This figure shows the results of the following questions: Q1. [1] “How much anxiety did you feel about your disease (ulcerative colitis or Crohn's disease) due to the outbreak of COVID-19?” The mean VAS score was 5.1 ± 2.5, indicating moderate anxiety. (B) Changes in VAS anxiety scores with the COVID-19 waves. The current study responses were obtained from the middle of the second COVID-19 wave to the end of the fourth wave in Japan. The VAS anxiety scores tended to change with the COVID-19 waves. VAS: visual analog scale. We added the figures on the change in the number of patients with SARS-CoV-2 infection in Japan according to the open data (by day) from "Understanding from Data—Information on New Coronavirus Infections" by the Ministry of Health, Labour and Welfare in Japan (by day) (https://covid19.mhlw.go.jp/extensions/public/index.html)

The primary outcome was the VAS score for disease-related anxiety during the COVID-19 pandemic (Q1 [1]). During this pandemic, the mean VAS score was 5.1 ± 2.5, which indicated moderate anxiety (Fig. 1A). No difference was observed between the VAS scores on anxiety among various Japanese regions. Figure 1B presents the changes in VAS anxiety scores alongside the changes in COVID-19 waves. Pearson's correlation coefficients indicated an increasing tendency in anxiety scores, a month after the number of infected persons per population increased; however, no significant correlations were found between the VAS scores for anxiety and the number of infected persons per population in the relevant month.

**Fig. 2:**
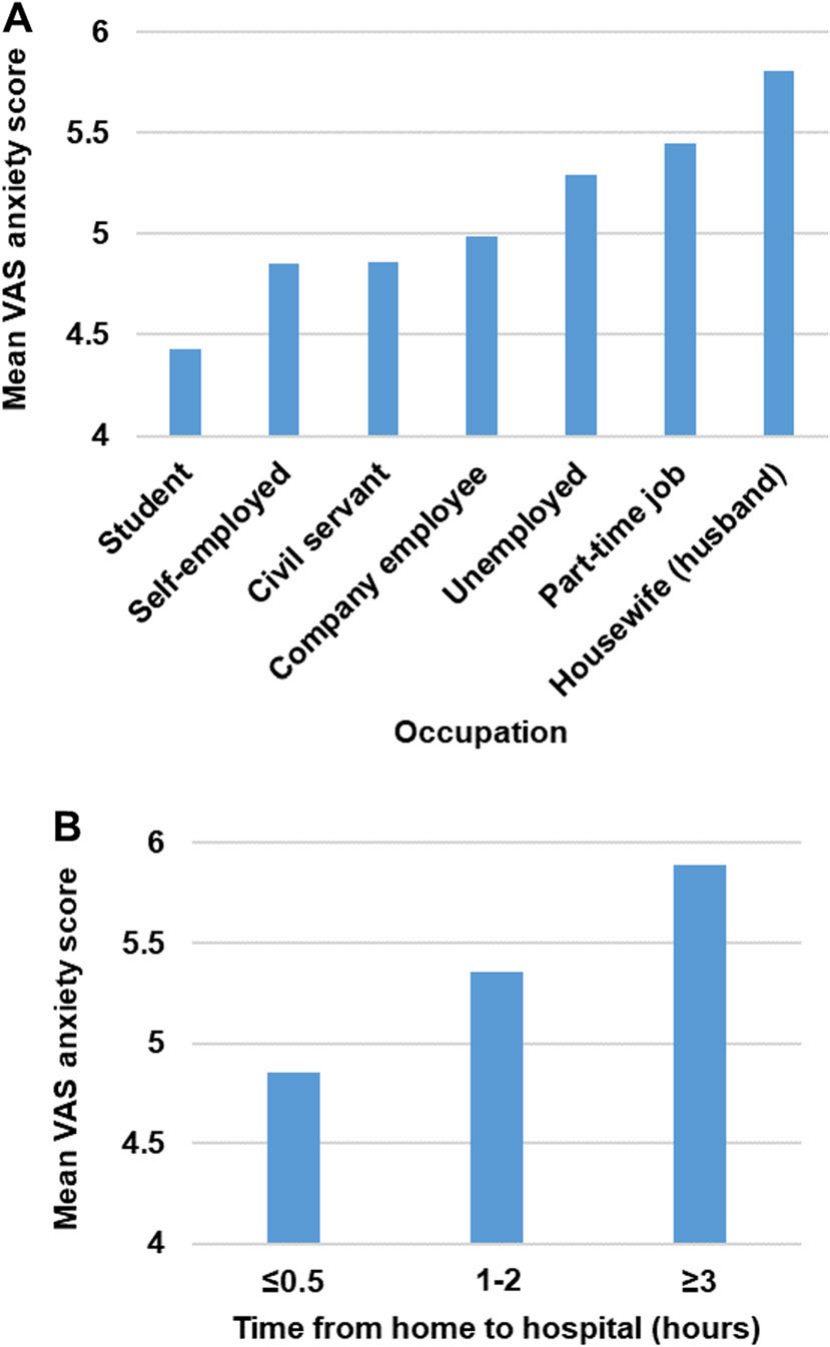
Factors associated with VAS anxiety scores. (A) The VAS score for disease-related anxiety during the COVID-19 pandemic by occupation. (B) The VAS score for disease-related anxiety during the COVID-19 pandemic for the time from home to hospital. VAS: visual analog scale

Univariate and multivariate analyses reported that gender (women), homemaker, arrival time at hospital from home, and usual means of commuting to the hospital (train) were significant factors for the VAS anxiety scores (Table 3). Similarly, the use of IBD medications, such as steroids, budesonide, thiopurine, tofacitinib, infliximab, ustekinumab, vedolizumab, and nutritional therapy were identified as significant factors (Tables 3, Table S1, Fig. 2A and 2B). In addition, both visiting the hospital and receiving a medical examination as scheduled and postponing or cancelling the examination significantly affected patients’ anxiety score. Interestingly, multivariate analysis demonstrated patients' anxiety scores decreased significantly after the start of vaccination (March 2021).

**Table 3:** Factors related to the anxiety experienced by Japanese patients with inflammatory bowel disease during the COVID-19 pandemic

Factors	%	Multivariate				
		Mean difference	Std. error	*P* value	95% Confidence interval	
					Lower bound	Upper bound
After the start of vaccination in Japan (after March 2021)		-0.26	0.13	0.04	-0.51	-0.01
Company employee vs Homemaker	44.7	-0.52	0.21	0.02	-0.94	-0.10
Student vs homemaker	5.1	-1.11	0.32	0.00	-1.73	-0.48
Civil servant vs homemaker	7.0	-0.64	0.27	0.02	-1.17	-0.10
Self-employed vs homemaker	7.0	-0.66	0.27	0.02	-1.19	-0.13
Female vs male	43.3	0.58	0.12	0.00	0.34	0.81
Time from home to hospital category	Continuous	0.39	0.10	0.00	0.19	0.59
Visiting the hospital and receiving a medical examination as scheduled	89.3	-0.46	0.17	0.01	-0.79	-0.12
Postponing or canceling the examinations	12.3	0.39	0.16	0.02	0.07	0.71
Usual means of commuting to the hospital—train	20.1	0.46	0.20	0.02	0.07	0.85
5-ASA	79.5	0.33	0.13	0.01	0.07	0.59
Oral steroid	6.9	0.68	0.21	0.00	0.28	1.09
Oral budesonide	1.8	0.80	0.40	0.05	0.01	1.59
Thiopurine	31.4	0.24	0.11	0.03	0.02	0.46
Tofacitinib	2.4	1.28	0.33	0.00	0.62	1.93
SASP suppositories	0.6	-1.41	0.71	0.05	-2.81	-0.01
Infliximab	19.1	0.47	0.16	0.00	0.17	0.78
Ustekinumab	8.4	0.41	0.20	0.04	0.02	0.81
Vedolizumab	5.9	0.51	0.22	0.02	0.07	0.95
Nutritional therapy	14.6	0.37	0.16	0.02	0.05	0.69

*ASA* 5-Aminosalicylic acid preparations

Only factors with *P* value < 0.05 in multivariate analysis have been described. The adjustment factors: part-time job vs homemaker; company employee vs homemaker; student vs homemaker; civil servant vs homemaker; self-employed vs homemaker; unemployed vs homemaker; age (every 10 years); female vs male; medical history category; surgical history category; married vs never married; Crohn's disease vs ulcerative colitis; usual interval between visits to the hospital category; time from home to hospital category; co-resident; stoma (artificial anus); attending a hospital near home; usual means of commuting to the hospital— walking or bicycling; usual means of commuting to the hospital—car; usual means of commuting to the hospital—bus; usual means of commuting to the hospital—train; usual means of commuting to the hospital—others; 5-aminosalicylic acid preparations; oral steroid; oral budesonide; thiopurine; tacrolimus; tofacitinib; pentasa suppositories; steroid suppositories; SASP suppositories; 5-aminosalicylic acid preparations enema; steroid enema (PREDONEMA®); steroid enema (STERONEMA®); budesonide enema; steroid suppositories; infliximab; adalimumab; golimumab; ustekinumab; vedolizumab; granulocyte apheresis therapy; nutritional therapy

**Fig. 3:**
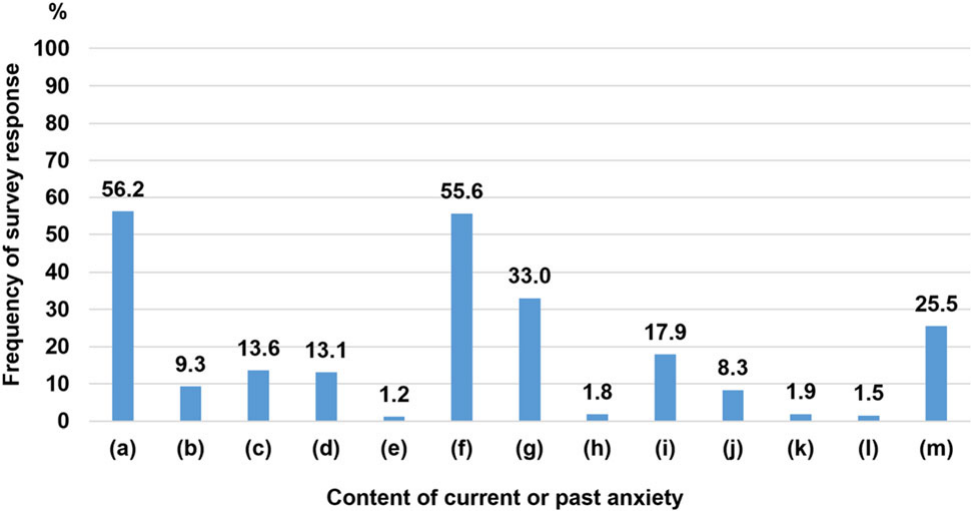
Contents of current or past anxiety during the COVID-19 pandemic. This figure is the result of the following questions: Q1. [2] “If you felt any anxiety in Q1 [1], what anxiety do you have? Please circle all that applies to your current or past concerns”. (a)–(m) shows the contents of the following anxiety: (a) I am worried about visiting a doctor because of the fear of contracting COVID-19. (b) I have a surplus of oral medication or can get a prescription by phone, but I feel uneasy when I have to visit a doctor for an intravenous drip or injection. (c) I am worried about visiting the hospital because I have to use public transportation. (d) The hospital is located far away, and I am worried about traveling to a city with prevalence of COVID19. (e) I am worried about visiting a doctor because I do not have masks or other goods that prevent infection. (f) I am worried that my disease itself may increase the possibility of contracting COVID-19. (g) I am worried that the medication used for treatment may increase the possibility of contracting COVID-19. (h) I am worried because the hospital has asked me to postpone my visit or examination. (i) I am worried that I will not be able to visit a hospital in the future due to hospital closures. (j) I feel anxious about paying for treatment because of a decrease in income. (k) I would like to see a doctor, but I am afraid of what people around me would think, so it is difficult for me to see a doctor. (l) It is difficult for me to see a doctor because I have to stay home with my child due to school closure, or because I am worried about infection when I leave my child somewhere else. (m) I am worried that I may pass COVID-19 to others. VAS: visual analog scale

Further, participants were asked about the contents of their anxiety (Fig. 3). The most common concerns reported were “I am worried about visiting a hospital because of the fear of contracting COVID-19 (56.2%),” “I am worried that my disease itself may increase the possibility of contracting COVID-19 (55.6%),” “I am worried that my medication might increase the possibility of contracting COVID-19 (33%),”, and "I am worried that I might pass on COVID-19 to others (25%)". Most of participants reported abdominal symptoms before the pandemic (Fig. S4), and their symptoms rarely worsened after the pandemic (Fig. S5). They believed that the pandemic did not strongly influence their own lives (Fig. S6).

### Changes in medical visits during the COVID-19 pandemic

**Fig. 4:**
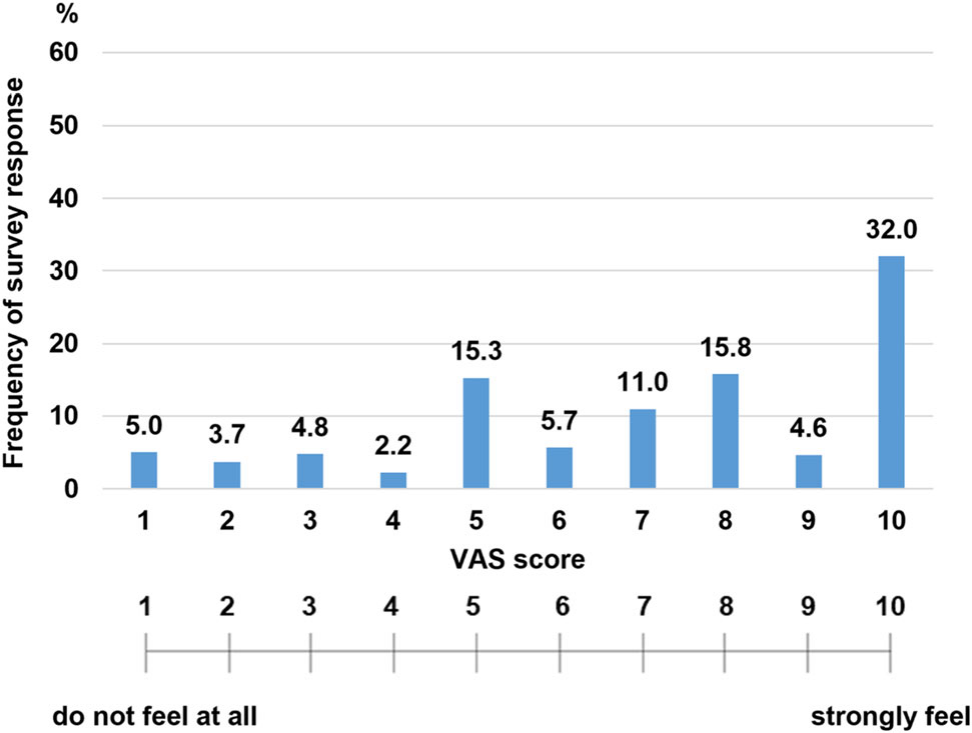
Participant responses indicated the need for a family doctor to manage patients with IBD amidst a pandemic. This figure represents the following question: Q2. [5] “How do you feel about the need for a family doctor specializing in IBD amidst an infectious disease such as COVID-19?” VAS: visual analog scale

The survey results for medical visits revealed that about 90% of the patients went to the hospital and received treatment as scheduled. Moreover, among the 9.9%, who could not visit the hospital or postponed their visit, 53.1% voluntarily postponed their visits, while 45.4% were advised to do so by their physicians. The usual interval between hospital visits was 1 month and 2–3 months for 26% and 73.5% of the patients, respectively (Table 1). The time taken to visit the hospital was < 30 min, 1–2 h, and > 3 h for 50.2%, 47.7%, and 2.1% patients, respectively (Table 1). Amidst the pandemic, only 5.5% of patients changed their mode of transportation to the hospital. For the question Q2 on medical visits (Medical visits. [5]) inquiring “How do you feel about the need for a family doctor specializing in IBD amidst an infectious disease pandemic, such as COVID-19?”, 32% of the participants reported experiencing a strong need (score 10), with 69% of the participants reporting a score of ≧ 6 points (Fig. 4).

### IBD medications during the COVID-19 pandemic

- The risk of IBD medications

Regarding the history of steroid use among the patients, 56.3%, 27.8%, and 8.9% had previously used, never used, and were currently using steroids, respectively.

Regarding the question whether steroids, immunomodulators, JAK inhibitors (tofacitinib), and biologics increase the risk of SARS-CoV-2 infection, about 50% of participants answered “I am not sure”. Participants determined the risk of infection related to these medications through several information sources (Fig. S7-9).

(b) Change in IBD treatments

During the pandemic, 97.5% of the patients continued oral medication and injections as recommended by physicians, 1.9% reduced the dose of medication, and 0.6% discontinued medication. The reasons for the patients’ reduced dose or discontinued medication were (1) stable abdominal symptoms (38.4%), (2) instructions from the attending physicians (31.5%), and (3) a higher risk of contracting infection due to medication (21.9%).

(c) Explanation of the continuation of IBD medication by physicians

A total of 42.6% received an explanation about the continuation of medication from their physicians, while 57.4% did not. Patients were highly satisfied with receiving the explanation (median VAS score is 10) (Fig. S10). Regarding anxiety after hearing the explanation, 45.7% responded that their anxiety remained the same; however, most of them scored < 5 on the VAS, suggesting that the explanation improved their anxiety (Fig. S11). Meanwhile, 91.5% of those who did not receive an explanation, did not ask their physicians about continuing medication.

### Information regarding prevention of COVID-19

Of the patients, 35.6% received information about the prevention of COVID-19 from their physicians, while 64.4% did not. Most patients were satisfied with the explanation from their physicians (median VAS score: 8) (Fig. S12 and S13), while 89.2% of the patients who did not receive an explanation did not intend to ask their physicians, and 10.2% wanted to ask them but could not.

### Endoscopic examination

Of the patients, 48.5% underwent the examination as scheduled, while 11.4% postponed it and 1.5% did not continue it. Among the patients who postponed or stopped endoscopic examination, 72.3% answered that their physicians asked them to refrain from the examination.

### Behavioral changes before and after the start of vaccination

We examined the behavioral changes before (February 2021) and after the start of vaccination (March 2021). Only the item "endoscopic examination" showed significant changes in behavior before and after the start of vaccination. However, we have not found any significant change for other items (means of transportation, visiting the hospital, reasons for postponing the visit, examinations, and continuation of treatment) (Table S2).

## Discussion

We conducted a large-scale survey among 3032 adult Japanese patients with IBD to demonstrate the impact of COVID-19 on these patients’ anxieties and health behaviors. The primary outcome was to evaluate patients’ disease-related anxiety during the COVID-19 pandemic using the VAS scores. We found that patients with IBD reported moderate disease-related anxiety; however, their VAS scores fluctuated with the COVID-19 waves. Additionally, the VAS score for anxiety tended to be associated with the number of infected people per population a month ago. Subsequently, the VAS scores for anxiety tended to increase one month after the number of infected persons per population increased. There were no significant regional differences in the VAS scores for anxiety in Japan. Univariate and multivariate analyses revealed that being a woman, a homemaker, hospital visit time and commuting to the hospital by train were factors associated with anxiety. We also identified the use of several IBD drugs, visiting the hospital and receiving a medical examination as scheduled, and postponing or cancelling the examination as factors related to patients’ anxiety. Of note, patients' anxiety scores decreased significantly after the start of vaccination.

Over 30% of the participants reported a strong need for community-based gastroenterologists during a pandemic situation similar to COVID-19. The current survey results of Japanese patients with IBD provide information regarding disease-related anxiety and behavioral changes during COVID-19 as well as the clinical issues encountered in the future management of patients with IBD.

The pandemic has greatly impacted the management of patients with IBD considering the enhanced risk of severe disease and death by COVID-19 with age and existing chronic disease [2]. Physicians are primarily concerned regarding the risk of COVID-19 in patients with IBD while determining their follow-up term, medical treatment course, and endoscopic assessment [15–19]. Further, many patients with IBD may lack information on COVID-19, and subsequently may be burdened by serious health concerns and anxiety.

This survey primarily focused on the disease-related anxiety in patients with IBD patients during the COVID-19 pandemic. The VAS scores revealed that participants experienced moderate anxiety, with the major concerns being the fear of (1) visiting a hospital due to the risk of contracting COVID-19 and (2) the disease itself increasing the risk of contracting COVID-19, consistent with previous reports [10–12]. Furthermore, we identified several factors associated with patients’ anxiety. Women reported higher anxiety scores than men and this tendency was consistent with that of the general population. Several studies have indicated that women are at a higher risk of developing anxiety and/or depressive symptoms [20, 21]; therefore, a pandemic situation might intensify their anxieties. Furthermore, the higher anxiety scores observed in unemployed and part-time workers may arise from concerns about their living costs if they contract COVID-19. Our results regarding the association of anxiety with the use of IBD medication and nutritional therapy have been investigated with other studies, which suggest that patients with IBD tend to overestimate the risks of biologics, tofacitinib, and thiopurine, despite contradictory evidence that there is no significant increase in the risk associated with these agents, except for when used in combination therapy [22, 23]. The Ministry of Health, Labour and Welfare has published the Japan IBD Task Force’s guidelines on patient care and medications for Japanese physicians on the websites of the Japan IBD research group and the Japanese Society for Inflammatory Bowel Disease [24]. However, the current survey results suggest that the explanation provided by doctors regarding the risks associated with drugs to patients may be inadequate. In Japan, nutritional therapy is provided in combination with several drugs. Accordingly, the higher anxiety levels among patients receiving nutritional therapy may result from high levels of concern about their nutritional status. Consistent with several studies on the concern regarding the risk of IBD medication [25, 26], our results revealed the importance of providing current and accurate information on COVID-19 to help mitigate medication-related anxiety and prevent inappropriate medication cessation. Regarding hospital visit, despite the pandemic situation, about 90% of the patients visited the hospital and received treatment on a regular basis. This result is not surprising since most of participants were attending a specialized IBD facility. In some Japanese regions, fewer physicians and medical staff are familiar with and experts in IBD treatment, thus, forcing many patients to go to specialized core hospitals. Therefore, most patients reported long duration for their hospital visit despite fear of contracting COVID-19; moreover, the impact of COVID-19 also increased patients’ need for a family doctor. Additionally, based on data regarding the behavioral changes before and after the start of vaccination, we found significantly behavioral changes in "endoscopic examination" before and after the start of vaccination. On the other hand, we did not find any significant changes for other items. The results suggest that physicians did not schedule endoscopic examinations, such as surveillance for patients with quiescent IBD, prior to the start of the vaccination during COVID-19 pandemic situation.

The results found that participants used their own thoughts, the internet, TV, and attending physicians as information sources, to evaluate the risk of infection from IBD itself and the corresponding medication. In a normal setting, patients with IBD ask physicians for information regarding IBD treatment and clinical problems. However, in this survey, Japanese patients with IBD primarily sought guidance, and information regarding COVID-19 from TV or Internet news. Several other surveys observed similar behavior [6, 7, 9]. The Japan IBD Task Force developed QR code to circulate a PDF file among patients with IBD, regarding COVID-19-related information required by these patients [27]. The results of this survey may reflect the limited number of patients who accessed this information, although it remains unclear whether they might feel that IBD physicians were not familiar with COVID-19. Combined with existing findings, the current survey data strongly suggest the need for a proactive, and interactive approach to IBD patient management.

This study has several limitations. First, the current study result interpretations are limited, as the primary outcome is measured only by a single item and the cross-sectional design restricts the interpretations of long-term effects of the pandemic. Future studies should employ a longitudinal study design using validated questionnaires. Second, since the sample only included patients with IBD over 16 years of age, we could not examine the anxiety among children with IBD and their parents. Third, the lack of a control group restricted comparisons of our findings with other adult populations’ findings. Fourth, we did not conduct a questionnaire survey during the fifth COVID-19 wave in Japan to avoid extending the study period, as we believe it was necessary to accumulate and analyze our data as soon as possible. Fifth, this survey was conducted within many specialized IBD centers. This particular setting may have influenced the need for community-based gastroenterologists.

In conclusion, we performed a large-scale data analysis to examine the effects of the COVID-19 pandemic on the anxiety and behavioral changes in Japanese patients with IBD. Subsequently, we reported the factors affecting anxiety, as well as the problems in the future management of patients with IBD. Future researchers and health-care professionals should continue to proactively inform patients about infectious diseases including COVID-19 and provide accurate corresponding information related to IBD care, thereby relieving patient anxiety as much as possible.

## Conflict of interest

Hiroshi Nakase reports receiving personal fees from AbbVie GK, Daiichi Sankyo, EA Pharma, Janssen, JIMRO, Kissei Pharmaceutical, Mitsubishi Tanabe Pharma, Mochida Pharmaceutical, Pfizer Inc., Takeda, and Gilead Sciences Inc., research grants from HOYA Pentax Medical, Pfizer Inc., Mitsubishi Tanabe Pharma Corporation, AbbVie GK, and Mochida Pharmaceutical Co., Ltd., and scholarship grants from AbbVie GK, EA Pharma, Mitsubishi Tanabe Pharma Corporation, and Nippon Kayaku. Takayuki Matsumoto reports receiving personal fees from Janssen, Tanabe-Mitsubishi Pharmaceutical, EA Pharma, and Takeda Pharmaceutical and scholarship grants from Nippon Kayaku and Tanabe-Mitsubishi Pharmaceutical. Minoru Matsuura reports receiving personal fees from Janssen Pharmaceutical K.K. and Takeda Pharmaceutical Co., Ltd. Katsuyoshi Matsuoka reports receiving personal fees from Mitsubishi Tanabe Pharma, AbbVie, Takeda Pharmaceutical, EA Pharma, Janssen Pharmaceutical, and Pfizer, research grants from Janssen Pharmaceutical, and scholarship grants from Mitsubishi Tanabe Pharma, AbbVie, EA Pharma, and Mochida Pharmaceutical. Shunji Ishihara reports receiving personal fees from Takeda Pharmaceutical Co., Ltd. and Mitsubishi Tanabe Pharma Corporation, and research grants from Janssen Pharmaceutical K.K. Fumihito Hirai reports receiving personal fees from AbbVie GK, EA Pharma Co., Ltd., Janssen Pharmaceutical K.K., Mochida Pharmaceutical Co., Ltd., Mitsubishi Tanabe Pharma Co., and Takeda Pharmaceutical Co., Ltd., research grants from Eli Lily Japan K.K., Janssen Pharmaceutical K.K., and AbbVie GK., and scholarship grants from AbbVie GK, EA Pharma Co., Ltd., Otsuka Pharmaceutical Co., Ltd., Kyorin Pharmaceutical Co., Ltd., Mochida Pharmaceutical Co., Ltd., and Mitsubishi Tanabe Pharma Co. Ken Takeuchi reports receiving personal fees from Mochida, AbbVie, Janssen pharma, Pfizer, and EA Pharma, and research grants from AbbVie, Takeda, Eli Lilly, Celgene, Shin Nippon Biomedical Laboratories, EA Pharma, Astra Zeneca, and Janssen pharma. Makoto Naganuma reports receiving personal fees from Takeda Pharmaceutical Co., Ltd., AbbVie GK, Mitsubishi Tanabe Pharma Corp, and Pfizer Co., Ltd., research grants from Mochida Pharmaceutical Co., Ltd., and scholarship grants from Takeda Pharmaceutical Co., Ltd., AbbVie GK, Mitsubishi Tanabe Pharma Corp, and Pfizer Co., Ltd. Kenji Watanabe reports receiving personal fees from, AbbVie Japan Co., Ltd., EA Pharma Co., Ltd., Pfizer Japan Inc., Takeda Pharmaceutical Co., Ltd., Mitsubishi Tanabe Pharma Corporation, Kyorin Pharmaceutical Co., Ltd., Mochida Pharmaceutical Co., Ltd., and Kissei Pharmaceutical Co., Ltd., research grants from EA Pharma Co., Ltd., Takeda Pharmaceutical Co., Ltd., and EP‐CRSU Co., Ltd., scholarship grants from AbbVie Japan Co., Ltd., EA Pharma Co., Ltd., Mitsubishi Tanabe Pharma Corporation, JIMRO Co., Ltd., and Nippon Kayaku Co., Ltd., and endowed chair from AbbVie Japan Co., Ltd., EA Pharma Co., Ltd., Mitsubishi Tanabe Pharma Corporation, ZERIA Pharmaceutical Co. Ltd., JIMRO Co., Ltd., Otsuka Pharmaceutical Factory, Inc., Asahi Kasei Medical Co., Ltd., and Mochida Pharmaceutical Co., Ltd. Satoshi Motoya reports receiving personal fees from AbbVie GK, Mitsubishi-Tanabe Pharma Corporation, Takeda Pharmaceutical Corporation, and Janssen Pharmaceutical KK., and scholarship grants from AbbVie GK and Janssen Pharmaceutical KK. Atsuo Maemoto reports receiving research grants from Gilead Sciences, Inc., Janssen Pharmaceutical K.K., Eli Lilly Japan K.K., Takeda Pharmaceutica Company, and Pfizer R&D Japan G.K. Hirotake Sakuraba reports receiving research grants from LAVIEPRE Co., Ltd., Yakult Honsha Co., Ltd., and Bristol-Myers Squibb Company, and scholarship grants from Eisai Co., Ltd. and Aomori Ai Co. Masayuki Saruta reports receiving personal fees from AbbVie GK, Janssen Pharmaceutical K.K., Mitsubishi Tanabe Pharma Co., Ltd., Takeda Pharmaceutical Co., Ltd., EA Pharma Co., Ltd., and Gilead Sciences K.K., research grants from EPS Corporation, and scholarship grants from Zeria Pharmaceutical Co., Ltd., Mochida Pharmaceutical Co., Ltd., EA Pharma Co., Ltd., and Kissei Pharmaceutical Co., Ltd. Hiroki Tanaka reports receiving personal fees from JIMRO Co., Ltd., AbbVie GK, EA Pharma Co., Ltd., Kyorin Pharmaceutical Co., Ltd., Mochida Pharmaceutical Co., Ltd., Kissei Pharmaceutical Co., Ltd., Eisai Co., Ltd., Mitsubishi Tanabe Pharma Corporation, Janssen Pharmaceutical K.K., Nikkiso Co., Ltd., and Takeda Pharmaceutical, and research grants from AbbVie GK, Janssen Pharmaceutical K.K., EA Pharma, and Takeda Pharmaceutical. Sakiko Hiraoka reports receiving personal fees from Janssen Pharmaceutical K.K., Mitsubishi Tanabe Pharma Corporation, AbbVie GK, and Takeda Pharmaceutical Co., Ltd. Tadakazu Hisamatsu reports receiving personal fees from EA pharma Co., Ltd., AbbVie GK, Celgene K.K., Janssen Pharmaceutical K.K., Pfizer Inc., Nichi-Iko Pharmaceutical Co., Ltd., Mitsubishi Tanabe Pharma Corporation, Kyorin Pharmaceutical Co., Ltd., JIMRO Co., Mochida Pharmaceutical Co., Ltd., and Takeda Pharmaceutical Co., Ltd., research grants from Alfresa Pharma Co., Ltd. and EA pharma Co., Ltd., and scholarship grants from Mitsubishi Tanabe Pharma Corporation, EA pharma Co., Ltd., AbbVie GK, JIMRO Co., Ltd., Zeria Pharmaceutical Co., Ltd., Daiichi-Sankyo, Kyorin Pharmaceutical Co., Ltd., Nippon Kayaku Co., Ltd., Takeda Pharmaceutical Co., Ltd., Pfizer Inc., and Mochida Pharmaceutical Co., Ltd.

## Supplementary Information

Below is the link to the electronic supplementary material.

Supplementary file1 (DOCX 250 KB)

## Data Availability

The data that support the findings of this study are available from the corresponding author upon reasonable request.

## Abbreviations

CD: Crohn’s disease
COVID-19: Coronavirus disease 2019
IBD: Inflammatory bowel disease
QOL: Quality of life
UC: Ulcerative colitis
VAS: Visual analog scale
