# Supplementary material for: Anxiety and behavioral changes in Japanese patients with inflammatory bowel disease due to COVID-19 pandemic: a national survey
Source: J Gastroenterol. 2023 Jan 6;58(3):205–16. doi: 10.1007/s00535-022-01949-6 (PMC9821342; doi:10.1007/s00535-022-01949-6)
Supplement: Supplementary file 1 — Supplementary file1 (DOCX 250 KB) [file 535_2022_1949_MOESM1_ESM.docx]

**Supplementary Material**

***Journal of Gastroenterology***

**Title: Anxiety and behavioral changes in Japanese patients with inflammatory bowel disease due to COVID-19 pandemic: A national survey.**

Hiroshi Nakase, Kohei Wagatsuma, Masanori Nojima, Takayuki Matsumoto, Minoru Matsuura, Hideki Iijima, Katsuyoshi Matsuoka, Naoki Ohmiya, Shunji Ishihara, Fumihito Hirai, Ken Takeuchi, Satoshi Tamura, Fukunori Kinjo, Nobuhiro Ueno, Makoto Naganuma, Kenji Watanabe, Rintaro Moroi, Nobuaki Nishimata, Satoshi Motoya, Koichi Kurahara, Sakuma Takahashi, Atsuo Maemoto, Hirotake Sakuraba, Masayuki Saruta, Keiichi Tominaga, Takashi Hisabe, Hiroki Tanaka, Shuji Terai, Sakiko Hiraoka, Hironobu Takedomi, Kazuyuki Narimatsu, Katsuya Endo, Masanao Nakamura, Tadakazu Hisamatsu. on behalf of J-DESIRE Group

**Correspondence to:** Hiroshi Nakase, M.D., Ph.D., AGAF

Department of Gastroenterology and Hepatology, Sapporo Medical University School of Medicine.

S-1, W-16, Chuo-ku, Sapporo, Hokkaido, 060-8543, Japan

Telephone: +81-11-611-2111

List of Supplementary Material

List of J-DESIRE Group members..................................................................................4

Questionnaire...............................................................................................................7

Supplementary Tables................................................................................................16

Supplementary Table S1. All adjustment factors associated with anxiety experienced by Japanese patients with inflammatory bowel disease during the COVID-19 pandemic................................................................................................................16

Supplementary Table S2. Behavioral changes before and after the start of vaccination...............................................................................................19

Supplementary Figures................................................................................................20

Supplementary Fig. S1 The age distribution of study participants (n=3032) ......20

Supplementary Fig. S2 Association between the monthly number of questionnaires and the COVID-19 waves ................................................................21

Supplementary Fig. S3 The number of questionnaires collected by region in Japan........................................................................................................................22

Supplementary Fig. S4 The symptoms of illness (ulcerative colitis or Crohn's disease) before the COVID-19 pandemic.................................................................23

Supplementary Fig. S5 The symptoms of illness (ulcerative colitis or Crohn's disease) after the COVID-19 pandemic....................................................................24

Supplementary Fig. S6 The impact of the COVID-19 pandemic on life...............25

Supplementary Fig. S7 Sources of information for the association between steroids and the risk of contracting COVID-19........................................................26

Supplementary Fig. S8 Sources of information for the association between immunomodulators or oral tacrolimus and the risk of COVID-19...........................27

Supplementary Fig. S9 Sources of information for the association between JAK inhibitors or biological agents and the risk of COVID-19........................................28

Supplementary Fig. S10 Satisfaction with the explanation of the association between COVID-19 and drugs................................................................................29

Supplementary Fig. S11 Changes in anxiety after receiving the explanation of the association between COVID-19 and drugs.............................................................30

Supplementary Fig. S12 Satisfaction with the explanation on prevention of COVID-19................................................................................................................31

Supplementary Fig. S13 Changes in anxiety after receiving the explanation on prevention of COVID-19.........................................................................................32

**List of J-DESIRE Group Members**

| **Study Team Member** | **Affiliation** |
| --- | --- |
| Sae Ohwada, M.D. | Department of Gastroenterology and Hepatology, Sapporo Medical University School of Medicine, Sapporo, Japan |
| Takehiro Hirano, M.D. | Department of Gastroenterology and Hepatology, Sapporo Medical University School of Medicine, Sapporo, Japan |
| Yoshihiro Yokoyama, M.D. | Department of Gastroenterology and Hepatology, Sapporo Medical University School of Medicine, Sapporo, Japan |
| Tsukasa Yamakawa, M.D. | Department of Gastroenterology and Hepatology, Sapporo Medical University School of Medicine, Sapporo, Japan |
| Yuki Hayashi, M.D. | Department of Gastroenterology and Hepatology, Sapporo Medical University School of Medicine, Sapporo, Japan |
| Tadashi Ichimiya, M.D. | Department of Gastroenterology and Hepatology, Sapporo Medical University School of Medicine, Sapporo, Japan |
| Tomoe Kazama, M.D. | Department of Gastroenterology and Hepatology, Sapporo Medical University School of Medicine, Sapporo, Japan |
| Daisuke Hirayama, M.D., Ph.D. | Department of Gastroenterology and Hepatology, Sapporo Medical University School of Medicine, Sapporo, Japan |
| Ayuko Hazumi, RN. | Division of Nursing, Sapporo Medical University Hospital, Sapporo, Japan |
| Ayumi Kadoya, B.S.N. | Division of Nursing, Sapporo Medical University Hospital, Sapporo, Japan |
| Saki Asakura, B.S.N. | Division of Nursing, Sapporo Medical University Hospital, Sapporo, Japan |
| Naomi Ebisawa, RN. | Division of Nursing, Sapporo Medical University Hospital, Sapporo, Japan |
| Shunichi Yanai, Ph.D. | Division of Gastroenterology, Department of Internal Medicine, Iwate Medical University, Shiwa, Japan |
| Daisuke Saito, M.D., Ph.D. | Department of Gastroenterology and Hepatology, Kyorin University School of Medicine, Mitaka, Japan |
| Shinichiro Shinzaki, M.D., Ph.D. | Department of Gastroenterology and Hepatology, Osaka University Graduate School of Medicine, Suita, Japan |
| Akihiro Yamada, M.D., Ph.D. | Division of Gastroenterology and Hepatology, Department of Internal Medicine, Toho University Sakura Medical Center, Sakura, Japan |
| Mitsuo Nagasaka, M.D., Ph.D. | Department of Gastroenterology, Fujita Health University School of Medicine, Toyoake, Japan |
| Kousaku Kawashima, M.D., Ph.D. | Department of Internal Medicine II, Shimane University Faculty of Medicine, Izumo, Japan |
| Nobuaki Kuno, M.D. | Department of Gastroenterology, Faculty of Medicine, Fukuoka University, Fukuoka, Japan |
| Yoshihiro Shimoyama, M.D., Ph.D. | Department of Gastroenterology, IBD Center, Tsujinaka Hospital Kashiwanoha, Kashiwa, Japan |
| Ken Sugimoto, M.D., Ph.D. | First Department of Medicine, Hamamatsu University School of Medicine, Hamamatsu, Japan |
| Nobufumi Uchima, M.D. | Center for Gastroenterology, Urasoe General Hospital, Urasoe, Japan |
| Mikihiro Fujiya, M.D., Ph.D. | Division of Metabolism and Biosystemic Science, Gastroenterology, and Hematology/Oncology, Department of Medicine, Asahikawa Medical University, Asahikawa, Japan |
| Norimasa Fukata, M.D., Ph.D. | The Third Department of Internal Medicine, Kansai Medical University, Hirakata, Japan |
| Yoko Yokoyama, M.D., Ph.D. | Center for Inflammatory Bowel Disease, Division of Internal Medicine, Hyogo College of Medicine, Nishinomiya, Japan |
| Atsushi Masamune, M.D., Ph.D. | Division of Gastroenterology, Tohoku University Hospital, Sendai, Japan |
| Yukinori Sameshima, Ph.D. | Department of Gastroenterology, Sameshima Hospital, Kagoshima, Japan |
| Ryosuke Kiyomori, M.D. | Division of Gastroenterology, Matsuyama Red Cross Hospital, Matsuyama, Japan |
| Tomoki Inaba, M.D., Ph.D. | Department of Gastroenterology, Kagawa Prefectural Central Hospital, Takamatsu, Japan |
| Takahiro Ito, M.D., Ph.D. | Inflammatory Bowel Disease Center, Sapporo Higashi Tokushukai Hospital, Sapporo, Japan |
| Hiroto Hiraga, M.D., Ph.D. | Department of Gastroenterology and Hematology, Hirosaki University Graduate School of Medicine, Hirosaki, Japan |
| Takahiko Toyonaga, M.D., Ph.D. | Department of Gastroenterology and Hepatology, Division of Internal Medicine, The Jikei University School of Medicine, Minato-ku, Japan |
| Takanao Tanaka, M.D. | Department of Gastroenterology, Dokkyo Medical University, Shimotsuga, Japan |
| Akihiro Koga, M.D., Ph.D. | Department of Gastroenterology, Fukuoka University Chikushi Hospital, Chikushino, Japan |
| Masanao Nasuno, M.D., Ph.D. | Sapporo IBD Clinic, Sapporo, Japan |
| Junji Yokoyama, M.D., Ph.D. | Division of Gastroenterology & Hepatology, Graduate School of Medical and Dental Sciences, Niigata University, Niigata, Japan |
| Eriko Yasutomi, M.D., Ph.D. | Department of Gastroenterology and Hepatology, Okayama University Graduate School of Medicine, Dentistry and Pharmaceutical Sciences, Okayama, Japan |
| Motohiro Esaki, M.D., Ph.D. | Division of Gastroenterology, Department of Internal Medicine, Faculty of Medicine, Saga University, Saga, Japan |
| Ryota Hokari, M.D., Ph.D. | Department of Internal Medicine, National Defense Medical College, Tokorozawa, Japan |
| Yuki Yoshino, M.D. | Division of Gastroenterology, Tohoku Medical and Pharmaceutical University, Sendai, Japan |

**Questionnaire**

Survey on COVID-19 for patients with inflammatory bowel disease

Please answer the following questions.

- **Demographics**

Age ( )

Gender ( Man / Woman )

Place of residence (prefecture: ) (city/town/village: )

Marital status ( Never married / Married )

Co-resident ( Yes / No )

Occupation (Company employee / Civil servant / Self-employed / Part-time job / Homemaker / Student / Unemployed)

Disease ( Ulcerative colitis / Crohn's disease )

Medical history ( ) years (Please indicate how many years have passed since you were diagnosed)

Surgical history ( ) times

Stoma (artificial anus) ( Yes ・ No )

- **Visit**

1. In addition to this hospital, do you visit any other hospital near your home for treatment of ulcerative colitis or Crohn's disease? ( Yes / No )
2. Usual interval between visits to the hospital ( within one month / 2-3 months / more than 4 months )
3. How long does it take you to get from your home to the hospital ( within 30 minutes / 1 to 2 hours / more than 3 hours )
4. Usual means of commuting to the hospital (walking or bicycling / car / bus / train / others)
5. Have you changed the way you go to the hospital during COVID-19 pandemic? ( Yes / No )
6. If you changed your method of hospital visit, please indicate your current method of hospital visit ( walk or bicycle / car / bus / train / others )

- **Current treatment status of your disease (ulcerative colitis or Crohn's disease)**

(Please exclude any treatment you received in the past but are not currently receiving.)

Please circle all that apply.

**Oral Medicine**

( ) 5-Aminosalicylic acid (ASA) preparations

( ) Steroids (Prednisone tablets)

( ) Budesonide

( ) Thiopurine drugs

( ) Tacrolimus

( ) Tofacitinib

**Suppository**

( ) Pentasa suppositories

( ) Steroid suppositories

( ) SASP suppositories

**Enema**

( ) 5-ASA enema

( ) Steroid enema (STERONEMA, PREDONEMA)

( ) Budesonide enema

**Injection**

( ) Steroid

( ) Infliximab

( ) Adalimumab

( ) Golimumab

( ) Ustekinumab

( ) Vedolizumab

Apheresis

( ) Granulocyte apheresis therapy

Nutritional therapy

( ) Yes

**Please circle all that apply to the following questions.**

**Q1. Concerns about SARS-CoV-2**

[1] How much anxiety did you feel about your disease (ulcerative colitis or Crohn's disease) due to the outbreak of SARS-CoV-2?


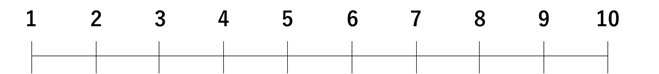


no anxiety (1) strong anxiety (10)

[2] If you felt any anxiety in question, what anxiety do you have?

Please circle all that apply to your current or past concerns, and if there are any others, please write them in the given space.

( ) I am worried about visiting a hospital because of the fear of contracting COVID-19 infection.

( ) I have a surplus of oral medication or can get a prescription by phone, but I feel uneasy when I have to visit a doctor for an intravenous drip or injection.

( ) I am worried about visiting the hospital because I have to use public transportation.

( ) The hospital is located far away, and I am worried about traveling to a city with prevalence of COVID-19.

( ) I am worried about visiting a doctor because I do not have masks or other goods that prevent infection.

( ) I am worried that my disease itself may increase the possibility of contracting COVID-19.

( ) I am worried that my medication might increase the possibility of contracting COVID-19.

( ) I am worried because the hospital has asked me to postpone my visit or examination.

( ) I am worried that I will not be able to visit a hospital in the future due to hospital closures.

( ) I feel anxious about paying for treatment due to a decrease in income.

( ) I would like to see a doctor, but I am afraid of what people around me would think, so it is difficult for me to see a doctor.

( ) It is difficult for me to see a doctor because I have to stay home with my child due to school closure, or because I am worried about infection when I leave my child somewhere else.

( ) I am worried that I might pass on COVID-19 to others.

[3]How were the symptoms of your illness (ulcerative colitis or Crohn's disease) before the COVID-19 pandemic?


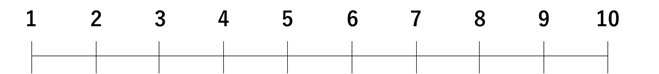


stable 　 unstable

(4) How were your symptoms of illness (ulcerative colitis or Crohn's disease) after the COVID-19 pandemic?


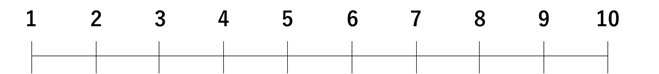


improved no change worsened

(5) Do you feel that the COVID-19 pandemic has affected your life?


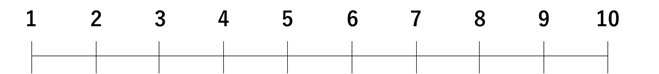


do not feel at all strongly feel

**Q2. Medical visits**

[1] Are you able to visit your doctor as scheduled? Please circle all that apply.

(a) I visit the hospital and receive medical examination as scheduled.

(b) I postponed my visit to the hospital, or extended the interval between visits.

(c) I went to the hospital. However, I did not have a medical examination and only received a prescription for medicine or an intravenous drip or injection as usual.

(d) I did not go to the hospital, but called for a prescription.

(e) I switched to another hospital near my house so that they could give me prescriptions, drops, and injections.

(f) I have not been able to see a doctor.

[2] If you could not see a doctor as planned ([1] b, c, d, e, f), what was the reason?

(a) My own decision (b) The hospital asked me to refrain from the examination

[3] Are you able to have an endoscopy and other examinations as scheduled?

(a) I am able to undergo the examinations as scheduled

(b) I postponed the examinations

(c) I cancelled the examinations

(d) I did not plan to perform the examinations

[4] This question is for those who did not perform the test as scheduled in question [3]. what was the reason for not undergoing an examination or for postponing it?

(a) My own judgment (b) The hospital asked me to refrain from the test

[5] How do you feel about the need for a family doctor specializing in IBD amidst an infectious disease pandemic such as COVID-19?


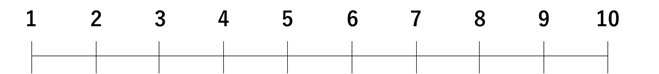


do not feel at all strongly feel

**Q3. Oral medication and injections**

[1] Have you continued your oral medication or injections during COVID-19?

(a) I continue to take oral medication and injections as instructed.

(b) I have reduced the amount or frequency of oral medication or injections.

(c) I have stopped taking oral medication or injections

[2] If you answered (b) or (c) in question [1], please circle all that apply, and if there are other reasons, please write them in the given column.

(a) Instructions of the attending physician

(b) I could not visit the hospital due to hospital’s reasons, and I ran out of oral medication/ I could not get an injection.

(c) I was worried about getting infected and did not visit the hospital. Therefore, I ran out of oral medication/ I could not get an injection.

(d) I was worried about getting infected, so I had oral medication prescribed over the phone, but could not get the injection because I did not visit the hospital.

(e) My own judgement that the medication increased the risk of infection reduced/stopped it.

(f) My abdominal symptoms were stable.

[3] We would like to ask those who answered (b) or (c) in question [1]. Have you told your doctor about it? (a) Yes (b) No

[4] Steroids

[4-1] Have you ever used steroids for your disease (ulcerative colitis or Crohn's disease)?

(a) I am currently using them

(b) I have used them in the past

(c) I have never used them

(d) I don't know

[4-2] Do you think that the use of these drugs increases the risk of infection with newer strains of coronavirus?


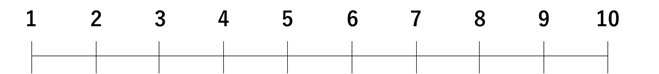


do not feel at all strongly feel

0: not sure

[4-3] This question is for those who answered 1 to 10 in [4-2]. What is the source of the information that made you think so? Please circle all that apply.

1. Only my own thoughts (b) Family or acquaintances (medical personnel)

(c) Family or acquaintances (non-medical personnel) (d) Primary doctor (e) Patient groups (f) Magazines (g) Newspapers (h) Internet (i) Television

[5] Immunomodulators and oral tacrolimus

[5-1] Have you ever used any of the following drugs for your disease (ulcerative colitis or Crohn's disease)?

(a) I am currently using it

(b) I have used it in the past

(c) I have never used it

(d) I don't know

[5-2] Do you think that the use of these drugs increases the risk of infection with new strains of coronavirus?


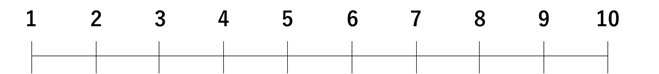


do not feel at all strongly feel

0: not sure

[5-3] This question is for those who answered 1 to 10 in [5-2]. What is the source of the information that made you think so? Please circle all that apply.

1. Only my own thoughts (b) Family or acquaintances (medical personnel) (c) Family

or acquaintances (non-medical personnel) (d) Primary doctor (e) Patient groups (f) Magazines (g) Newspapers (h) Internet (i) Television

[6] JAK inhibitors, biological agents

[6-1] Have you ever used them for your disease (ulcerative colitis or Crohn's disease)?

(a) I am currently using them

(b) I have used them in the past

(c) I have never used them

(d) I don't know

[6-2] Do you think that the use of these drugs increases the risk of infection with new strains of coronavirus?


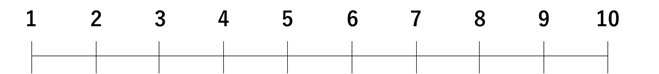


do not feel at all strongly feel

0: not sure

[6-3] This question is for those who answered 1 to 10 in [6-2]. What is the source of the information that made you think so? Please circle all that apply.

1. Only my own thoughts (b) Family or acquaintances (medical personnel) (c) Family

or acquaintances (non-medical personnel) (d) Primary doctor (e) Patient groups (f) Magazines (g) Newspapers (h) Internet (i) Television

**Q4. About the disease (ulcerative colitis or Crohn's disease)**

[1] Do you think that having ulcerative colitis or Crohn's disease itself increases the risk of infection with new strains of coronavirus?


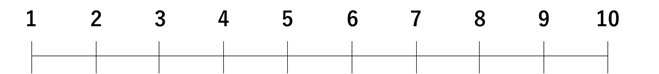


do not feel at all strongly feel

[2] What is the source of the information that made you think like [1]? Please circle all that apply.

1. Only my own thoughts (b) Family or acquaintances (medical personnel) (c) Family

or acquaintances (non-medical personnel) (d) Primary doctor (e) Patient groups (f) Magazines (g) Newspapers (h) Internet (i) Television

**Q5. Explanation of prevention of SARS-CoV-2**

[1] Did your doctor provide you with information on how to prevent new coronavirus infection?

(a) Yes (b) No

[2] This question is for those who answered (a) in [1]. Were you satisfied with the explanation?


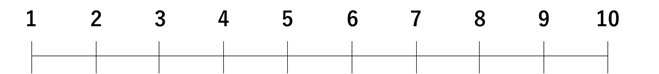


not satisfied satisfied

[3] If you answered (a) in [1] above, please answer. How did your anxiety change after listening to the explanation?


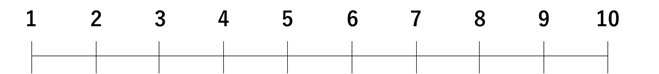


reduced no change increased

(4) This question is for those who answered (b) in [1].

(a) I asked physicians but they did not tell me.

(b) I wanted to ask, but could not.

(c) I did not ask my doctor because I did not intend to ask him/her.

**Q6. Explanation of the relationship between new strains of coronavirus and drugs**

[1] Did your doctor tell you whether you could continue the current medication during the COVID-19 pandemic?

(a) Yes (b) No

[2] This question is for those who answered (a) in [1]. Were you satisfied with the explanation?


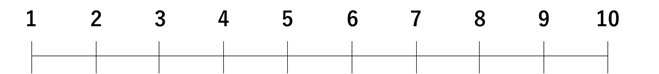


not satisfied satisfied

[3] If you answered (a) in [1], please answer this question. How did your anxiety change after hearing the explanation?


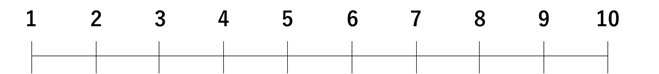


reduced no change increased

[4] This question is for those who answered (b) in [1].

(a) I asked physicians but they did not tell me.

(b) I wanted to ask, but could not.

(c) I did not ask my doctor because I did not intend to ask him/her.

**Thank you for your cooperation.**

**Supplementary Tables**

**Supplementary Table S1. All adjustment factors associated with anxiety experienced by Japanese patients with inflammatory bowel disease during the COVID-19 pandemic.**

|  |  | **Univariate** | | | | |  | **Multivariate** | | | | |
| --- | --- | --- | --- | --- | --- | --- | --- | --- | --- | --- | --- | --- |
| Factors | % | Mean difference | Std. Error | P value | 95% Confidence Interval | |  | Mean difference | Std. Error | P value | 95% Confidence Interval | |
|  |  |  |  |  | Lower Bound | Upper Bound |  |  |  |  | Lower Bound | Upper Bound |
| Intercept |  |  |  |  |  |  |  | 4.158 | 0.568 | <0.001 | 3.045 | 5.271 |
| Part-time job vs Homemaker | 14.3 | -0.355 | 0.193 | 0.066 | -0.734 | 0.023 |  | -0.273 | 0.219 | 0.212 | -0.702 | 0.156 |
| Company employee vs Homemaker | 44.7 | -0.813 | 0.165 | <0.001 | -1.136 | -0.490 |  | -0.523 | 0.207 | 0.012 | -0.930 | -0.116 |
| Student vs Homemaker | 5.1 | -1.375 | 0.252 | <0.001 | -1.869 | -0.881 |  | -1.127 | 0.312 | <0.001 | -1.738 | -0.515 |
| Civil servant vs Homemaker | 7.0 | -0.941 | 0.229 | <0.001 | -1.389 | -0.492 |  | -0.630 | 0.269 | 0.019 | -1.159 | -0.102 |
| Self-employed vs Homemaker | 7.0 | -0.953 | 0.229 | <0.001 | -1.402 | -0.504 |  | -0.669 | 0.263 | 0.011 | -1.185 | -0.152 |
| Unemployed vs Homemaker | 12.2 | -0.512 | 0.200 | 0.011 | -0.905 | -0.119 |  | -0.283 | 0.244 | 0.245 | -0.762 | 0.195 |
| Homemaker | 9.5 | Ref. | . | . | . | . |  | Ref. | . | . | . | . |
| Age (every 10 years) | continuous | 0.023 | 0.031 | 0.454 | -0.038 | 0.084 |  | 0.006 | 0.046 | 0.892 | -0.085 | 0.097 |
| Female vs male | 43.3 | 0.684 | 0.093 | <0.001 | 0.502 | 0.866 |  | 0.568 | 0.120 | <0.001 | 0.333 | 0.802 |
| Medical history category | continuous | 0.067 | 0.031 | 0.030 | 0.007 | 0.128 |  | -0.013 | 0.039 | 0.736 | -0.090 | 0.064 |
| Surgical history category | continuous | 0.138 | 0.049 | 0.005 | 0.043 | 0.233 |  | 0.004 | 0.069 | 0.952 | -0.130 | 0.139 |
| Married vs Never married | 59.2 | -0.063 | 0.095 | 0.507 | -0.248 | 0.123 |  | -0.148 | 0.133 | 0.267 | -0.410 | 0.113 |
| CD vs UC | 39.4 | 0.278 | 0.095 | 0.004 | 0.091 | 0.464 |  | 0.185 | 0.152 | 0.224 | -0.113 | 0.482 |
| Usual interval between visits to the hospital category | continuous | -0.424 | 0.104 | <0.001 | -0.628 | -0.220 |  | -0.198 | 0.117 | 0.091 | -0.428 | 0.032 |
| Time from home to hospital category | continuous | 0.506 | 0.086 | <0.001 | 0.337 | 0.675 |  | 0.354 | 0.099 | <0.001 | 0.160 | 0.549 |
| Co-resident | 83.8 | 0.223 | 0.126 | 0.077 | -0.024 | 0.471 |  | 0.267 | 0.150 | 0.075 | -0.027 | 0.561 |
| Stoma (artificial anus) | 4.9 | 0.401 | 0.217 | 0.065 | -0.025 | 0.826 |  | 0.232 | 0.260 | 0.372 | -0.277 | 0.741 |
| Attending a hospital near home | 7.1 | 0.431 | 0.180 | 0.017 | 0.078 | 0.784 |  | 0.313 | 0.203 | 0.122 | -0.084 | 0.710 |
| Usual means of commuting to the hospital: Walking or Bicycling | 7.8 | -0.369 | 0.174 | 0.034 | -0.711 | -0.027 |  | -0.094 | 0.234 | 0.686 | -0.553 | 0.364 |
| Usual means of commuting to the hospital: Car | 72.5 | -0.118 | 0.104 | 0.258 | -0.323 | 0.087 |  | 0.256 | 0.206 | 0.213 | -0.147 | 0.659 |
| Usual means of commuting to the hospital: Bus | 10.5 | 0.492 | 0.153 | 0.001 | 0.192 | 0.791 |  | 0.155 | 0.182 | 0.394 | -0.202 | 0.512 |
| Usual means of commuting to the hospital: Train | 20.1 | 0.499 | 0.116 | <0.001 | 0.272 | 0.727 |  | 0.430 | 0.196 | 0.029 | 0.045 | 0.815 |
| Usual means of commuting to the hospital: Others | 2.8 | 0.314 | 0.291 | 0.281 | -0.257 | 0.885 |  | 0.203 | 0.330 | 0.537 | -0.443 | 0.850 |
| 5-ASA | 79.5 | 0.220 | 0.115 | 0.056 | -0.006 | 0.446 |  | 0.313 | 0.129 | 0.015 | 0.060 | 0.566 |
| Oral steroid | 6.9 | 0.751 | 0.183 | <0.001 | 0.392 | 1.110 |  | 0.650 | 0.204 | 0.001 | 0.250 | 1.051 |
| Oral Budesonide | 1.8 | 1.236 | 0.363 | 0.001 | 0.525 | 1.947 |  | 0.656 | 0.388 | 0.091 | -0.104 | 1.417 |
| Thiopurine | 31.4 | 0.275 | 0.100 | 0.006 | 0.079 | 0.471 |  | 0.224 | 0.112 | 0.045 | 0.005 | 0.443 |
| Tacrolimus | 1.0 | 1.174 | 0.473 | 0.013 | 0.247 | 2.102 |  | 0.609 | 0.521 | 0.243 | -0.414 | 1.631 |
| Tofacitinib | 2.4 | 1.023 | 0.302 | 0.001 | 0.431 | 1.615 |  | 1.150 | 0.328 | <0.001 | 0.506 | 1.793 |
| Pentasa suppositories | 12.0 | -0.002 | 0.143 | 0.986 | -0.283 | 0.278 |  | 0.159 | 0.161 | 0.322 | -0.156 | 0.474 |
| Steroid suppositories | 0.8 | 0.527 | 0.543 | 0.331 | -0.537 | 1.592 |  | 0.254 | 0.542 | 0.640 | -0.809 | 1.316 |
| SASP suppositories | 0.6 | -0.646 | 0.617 | 0.295 | -1.856 | 0.564 |  | -1.396 | 0.716 | 0.051 | -2.800 | 0.008 |
| 5-ASA enema | 4.5 | 0.547 | 0.225 | 0.015 | 0.106 | 0.988 |  | 0.258 | 0.253 | 0.308 | -0.238 | 0.754 |
| Steroid enema (PREDONEMA®) | 0.6 | 0.999 | 0.584 | 0.087 | -0.146 | 2.143 |  | 0.747 | 0.601 | 0.213 | -0.430 | 1.925 |
| Steroid enema (STERONEMA®) | 0.3 | 0.444 | 0.847 | 0.600 | -1.217 | 2.105 |  | -0.824 | 1.040 | 0.428 | -2.863 | 1.215 |
| Budesonide enema | 6.5 | 0.252 | 0.189 | 0.182 | -0.119 | 0.624 |  | 0.178 | 0.216 | 0.410 | -0.245 | 0.600 |
| Steroid suppositories | 1.2 | 0.242 | 0.420 | 0.565 | -0.582 | 1.065 |  | -0.291 | 0.455 | 0.522 | -1.183 | 0.601 |
| Infliximab | 19.1 | 0.363 | 0.118 | 0.002 | 0.132 | 0.593 |  | 0.371 | 0.152 | 0.015 | 0.072 | 0.669 |
| Adalimumab | 12.8 | 0.065 | 0.139 | 0.638 | -0.207 | 0.338 |  | 0.180 | 0.170 | 0.289 | -0.153 | 0.512 |
| Golimumab | 2.3 | 0.129 | 0.314 | 0.681 | -0.486 | 0.744 |  | -0.071 | 0.341 | 0.835 | -0.739 | 0.597 |
| Ustekinumab | 8.4 | 0.394 | 0.167 | 0.018 | 0.067 | 0.722 |  | 0.346 | 0.196 | 0.077 | -0.038 | 0.730 |
| Vedolizumab | 5.9 | 0.352 | 0.197 | 0.074 | -0.034 | 0.737 |  | 0.459 | 0.220 | 0.037 | 0.027 | 0.890 |
| Granulocyte apheresis therapy | 1.1 | 0.778 | 0.437 | 0.075 | -0.079 | 1.636 |  | 0.323 | 0.510 | 0.527 | -0.677 | 1.322 |
| Nutritional therapy | 14.6 | 0.611 | 0.136 | <0.001 | 0.344 | 0.877 |  | 0.358 | 0.160 | 0.026 | 0.044 | 0.673 |

ASA: 5-Aminosalicylic acid preparations, CD: Crohn's disease, UC: Ulcerative colitis

**Supplementary Table S2. Behavioral changes before and after the start of vaccination**

|  | **Before the start of vaccination in Japan (Before February 2021)** | **After the start of vaccination in Japan (After March 2021)** |  |
| --- | --- | --- | --- |
| **Q2. [3] Are you able to have an endoscopy and other examinations as scheduled?** | **n (%)** | **n (%)** | **P value†** |
| I am able to undergo the examinations as scheduled. | 1029  (45.1%) | 372  (61.3%) | <0.001 |
| I postponed the examinations. /  I cancelled the examinations. | 299  (13.1%) | 74  (12.2%) |  |
| I did not plan to perform the examinations. | 956  (41.9%) | 161  (26.5%) |  |

† Chi-squared test

**Supplementary Figures**

**Supplementary Fig. S1** The age distribution of study participants (n=3032)


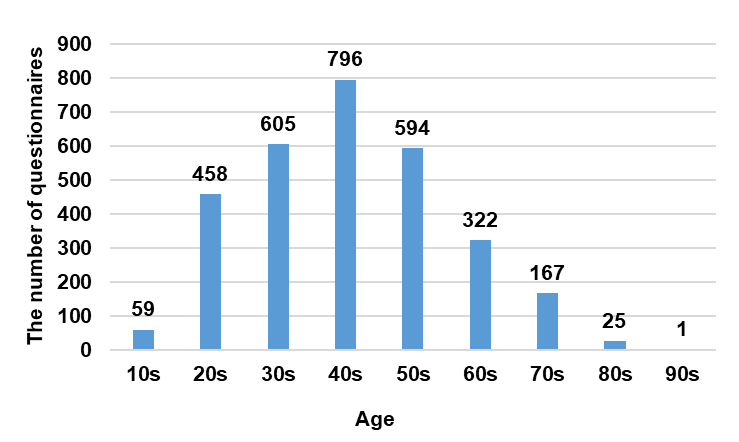


The median age of the participants was 44 years (range: 16-92 years).

**Supplementary Fig. S2** Association between the monthly number of questionnaires and the COVID-19 waves

**
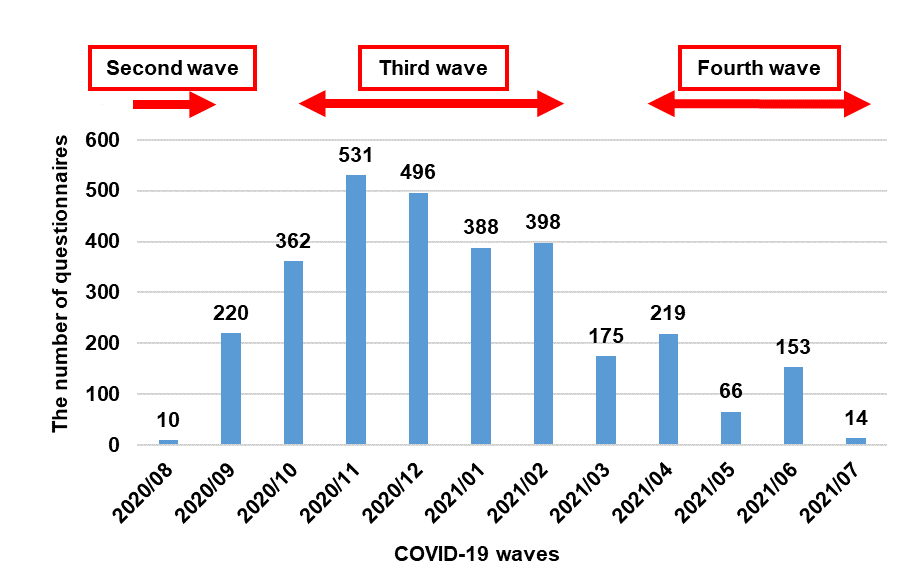
**

The study period spanned between March 2020 and June 2021. The number of questionnaires indicates the month for sending the questionnaires.

**Supplementary Fig. S3** The number of questionnaires collected by region in Japan

**
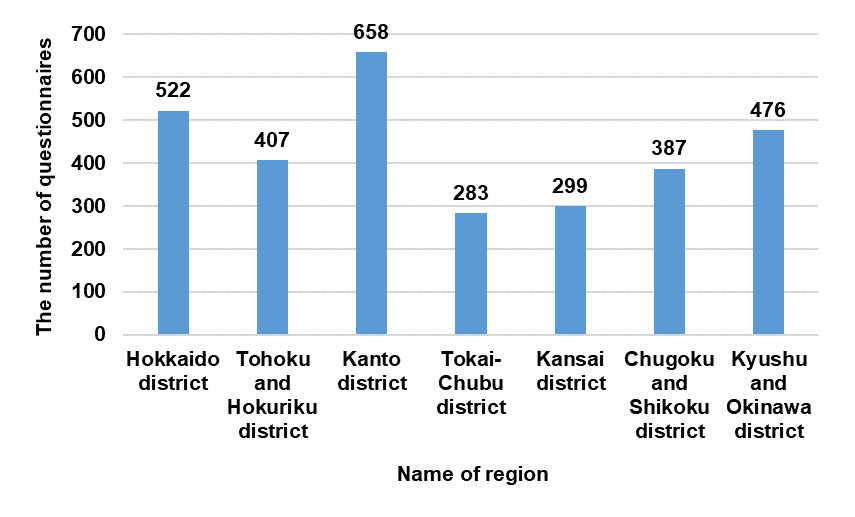
**

The survey was conducted across various Japanese regions, and the number of questionnaires varied by region.

**Supplementary Fig. S4** The symptoms of illness (ulcerative colitis or Crohn's disease) before the COVID-19 pandemic


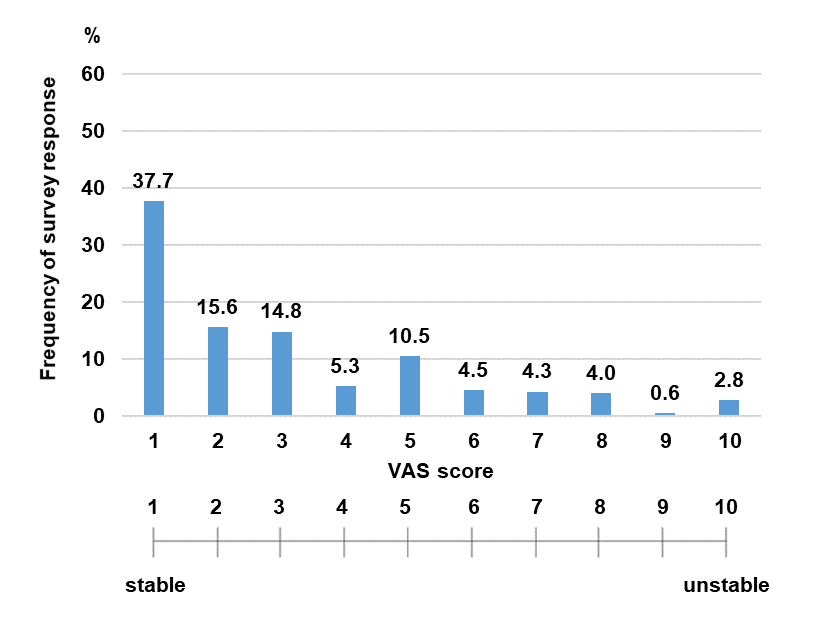


This figure is the result of the following questions: Q1. [3] “How were the symptoms of your illness (ulcerative colitis or Crohn's disease) before the COVID-19 pandemic?”. VAS: Visual Analog Scale.

**Supplementary Fig. S5** The symptoms of illness (ulcerative colitis or Crohn's disease) after the COVID-19 pandemic

**
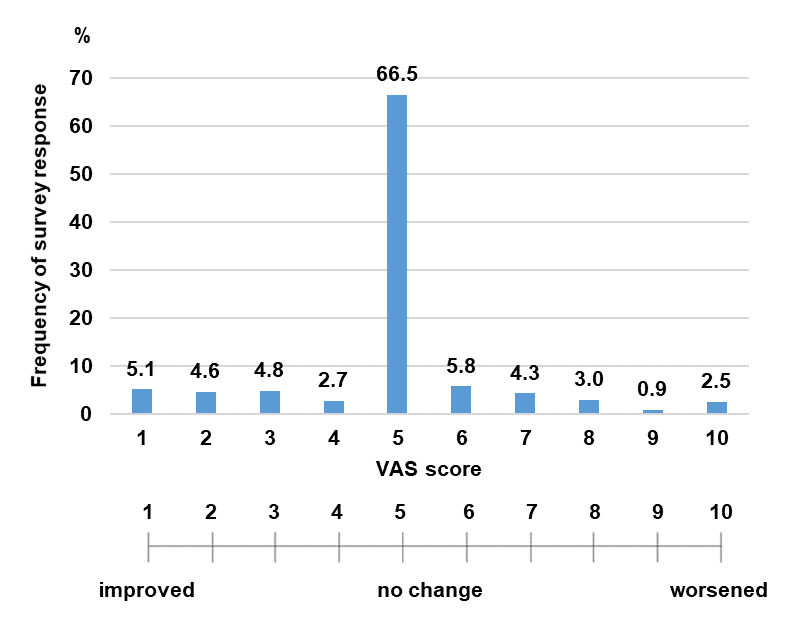
**

This figure is the result of the following questions: Q1. [4] “How were your symptoms of illness (ulcerative colitis or Crohn's disease) after the COVID-19 pandemic?”. VAS: Visual Analog Scale.

**Supplementary Fig. S6** The impact of the COVID-19 pandemic on life


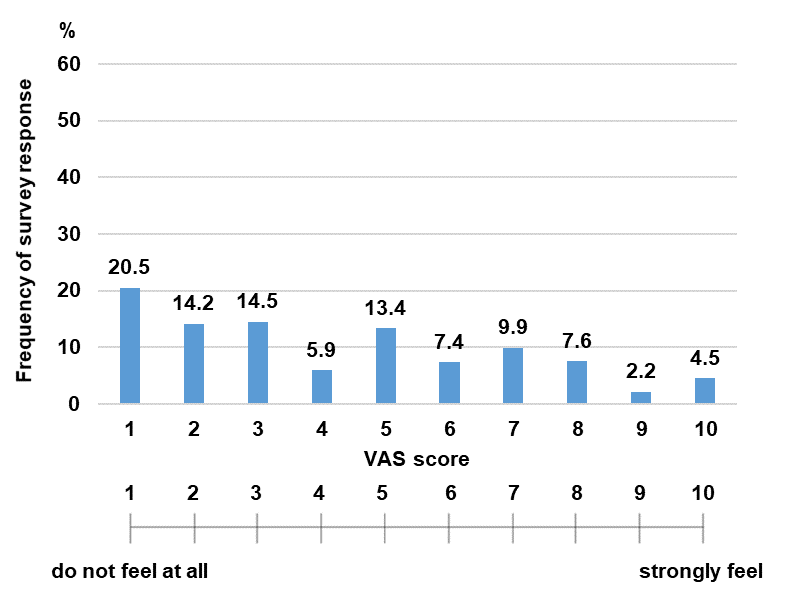


This figure is the result of the following questions: Q1. [5] “Do you feel that the COVID-19 pandemic has affected your life?”. VAS: Visual Analogue Scale.

**Supplementary Fig. S7** Sources of information for the association between steroids and the risk of contracting COVID-19


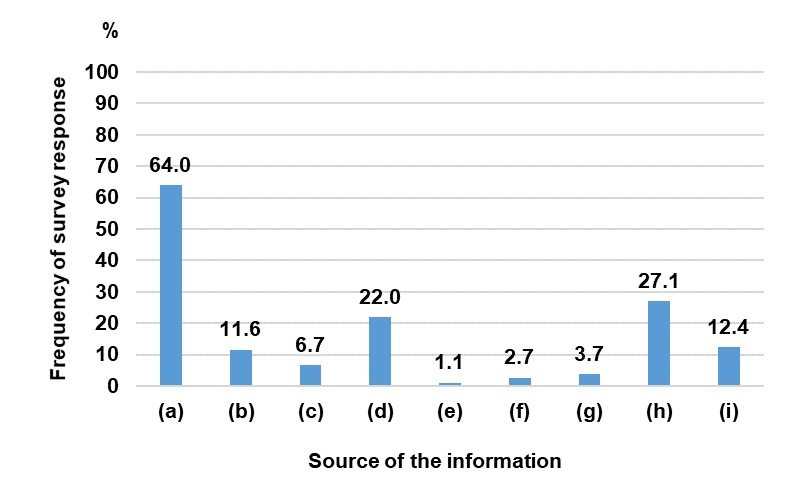


This figure is the result of the following question: Q3. [4-3] “This question is for those who answered 1 to 10 in [4-2] “Do you think that the use of steroids increases the risk of infection with newer strains of coronavirus?”. What is the source of the information that made you think so? Please circle all that apply. (a)-(i) shows the contents of the following anxiety: (a) Only my own thoughts; (b) Family or acquaintances (medical personnel); (c) Family or acquaintances (non-medical personnel); (d) Primary doctor; (e) Patient groups; (f) Magazines; (g) Newspapers; (h) Internet; and (i) Television.

**Supplementary Fig. S8** Sources of information for the association between immunomodulators or oral tacrolimus and the risk of COVID-19


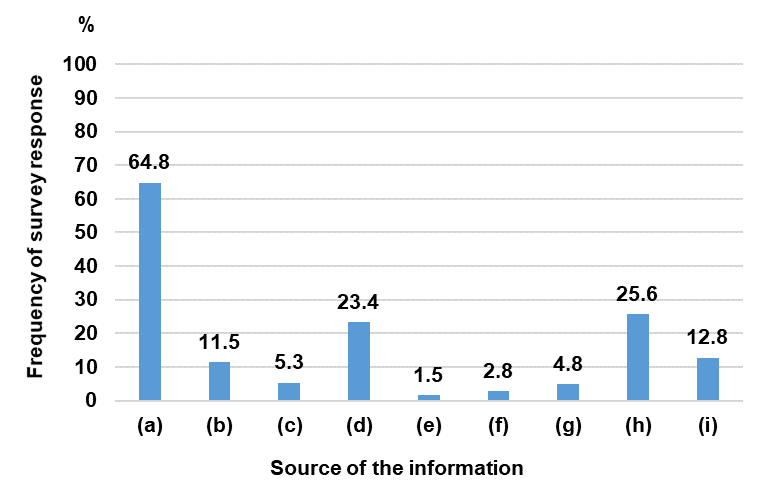


This figure is the result of the following question: Q3. [5-3] “This question is for those who answered 1 to 10 in [5-2] “Do you think that the use of Immunomodulators or oral tacrolimus increases the risk of infection with newer strains of coronavirus?”. What is the source of the information that made you think so? Please circle all that apply. (a)-(i) shows the contents of the following anxiety: (a) Only my own thoughts; (b) Family or acquaintances (medical personnel); (c) Family or acquaintances (non-medical personnel); (d) Primary doctor; (e) Patient groups; (f) Magazines; (g) Newspapers; (h) Internet; and (i) Television.

**Supplementary Fig. S9** Sources of information for the association between JAK inhibitors or biological agents and the risk of COVID-19


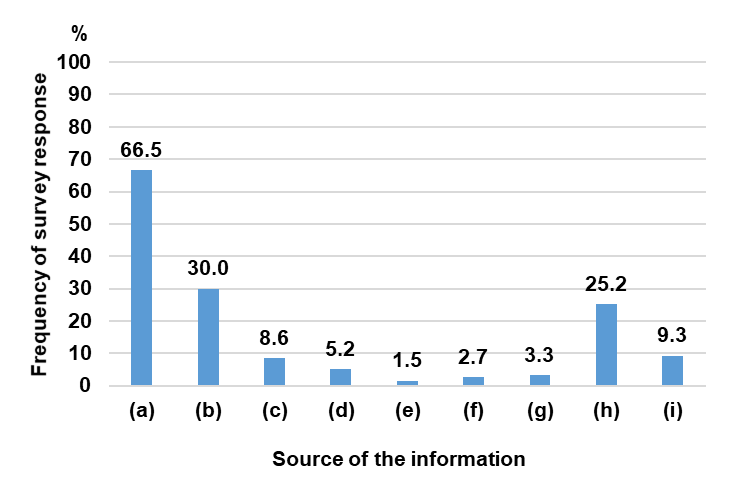


This figure is the result of the following question: Q3. [6-3] “This question is for those who answered 1 to 10 in [6-2] “Do you think that the use of JAK inhibitors or biological agents increases the risk of infection with newer strains of coronavirus?”. What is the source of the information that made you think so? Please circle all that apply. (a)-(i) shows the contents of the following anxiety: (a) Only my own thoughts; (b) Primary doctor; (c) Family or acquaintances (medical personnel); (d) Family or acquaintances (non-medical personnel); (e) Patient groups; (f) Magazines; (g) Newspapers; (h) Internet; and (i) Television. Missing values for each item were excluded from the denominator.

**Supplementary Fig. S10** Satisfaction with the explanation of the association between COVID-19 and drugs


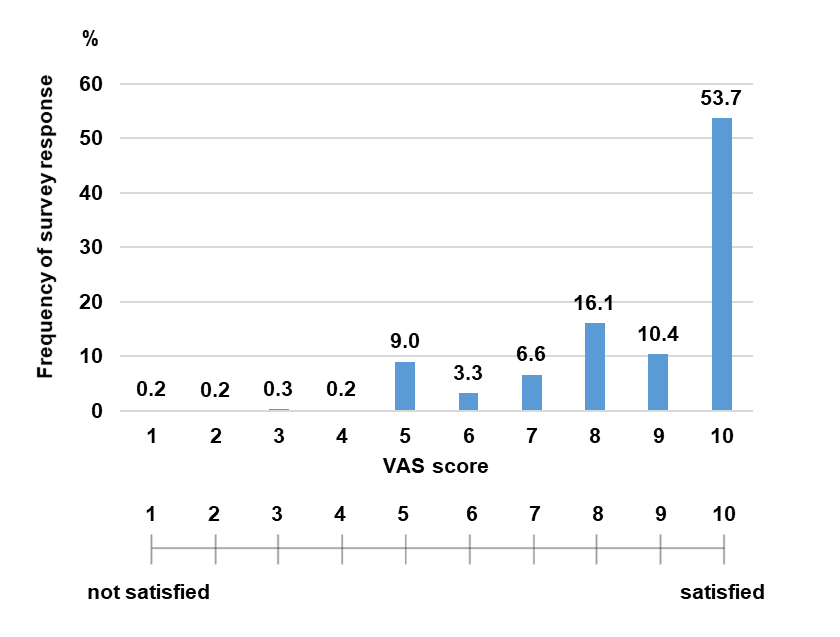


This figure is the result of the following questions: Q6. [2] “This question is for those who answered Yes in Q6. [1] “Did your doctor tell you whether you could continue the current medication during the COVID-19 pandemic?”” “Were you satisfied with the explanation?”. VAS: Visual Analog Scale.

**Supplementary Fig. S11** Changes in anxiety after receiving the explanation of the association between COVID-19 and drugs


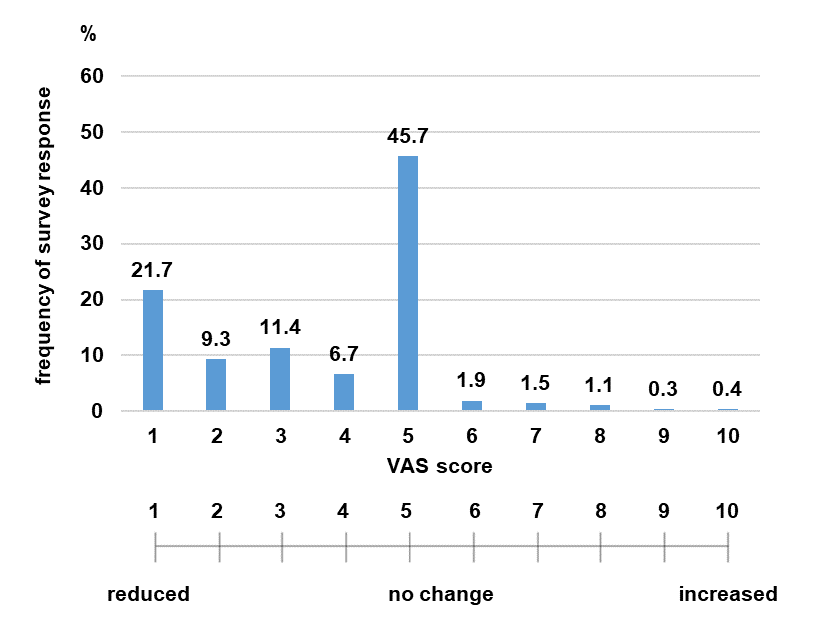


This figure is the result of the following questions: Q6. [3] “If you answered Yes in Q6. [1] “Did your doctor tell you whether you could continue the current medication during the COVID-19 pandemic?” above, please answer. “How did your anxiety change after hearing the explanation?”. VAS: Visual Analog Scale.

**Supplementary Fig. S12** Satisfaction with the explanation on prevention of COVID-19


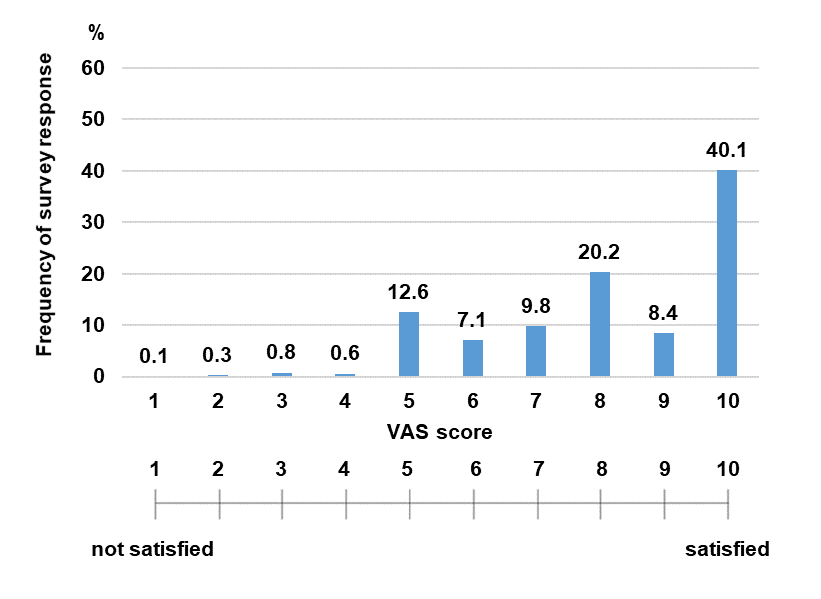


This figure is the result of the following questions: Q5. [2] This question is for those who answered Yes in Q5. [1] “Did your doctor provide you with information on how to prevent new coronavirus infection?” “Were you satisfied with the explanation?” VAS: Visual Analog Scale.

**Supplementary Fig. S13** Changes in anxiety after receiving the explanation on prevention of COVID-19


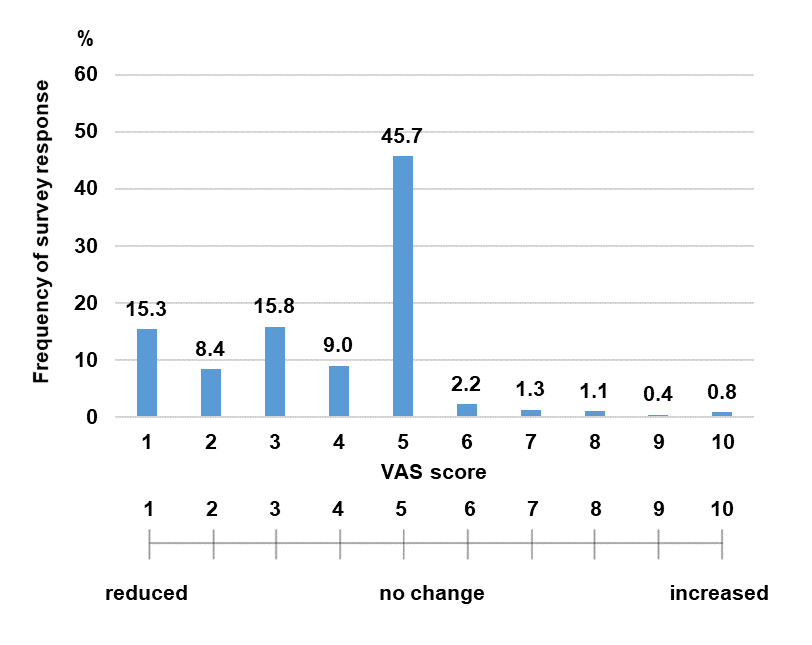


This figure is the result of the following questions: Q5. [3] If you answered Yes in Q5. [1] “Did your doctor provide you with information on how to prevent new coronavirus infection?” above, please answer. “How did your anxiety change after listening to the explanation?” VAS: Visual Analog Scale.
